# Geographic differentiation of agritourism activities in Poland vs. cultural and natural attractiveness of destinations at district level

**DOI:** 10.1371/journal.pone.0222576

**Published:** 2019-09-20

**Authors:** Arkadiusz Sadowski, Monika Małgorzata Wojcieszak

**Affiliations:** Department of Economics and Economic Policy in Agribusiness, Faculty of Economics and Social Sciences, Poznan University of Life Sciences, Poznań, Poland; Politecnico di Milano, ITALY

## Abstract

One of the trends in today’s tourism sector is the development of environmentally-friendly tourism activities which rely on natural resources of cultural heritage and on biodiversity. This is definitely the case for agritourism, a form of rural tourism. The purpose of this paper is to identify the development aspects of Polish agritourism with particular emphasis on natural and cultural attractiveness. To demonstrate the relationship between agritourism activities of Polish farms and the cultural and natural attractiveness, the Hellwig’s synthetic development indicator was used. As shown by research, the cultural and natural attractiveness of a destination is an important exogenous development factor. Another finding was that the intensified efforts undertaken by the farmers to access EU funds were not focused on areas with valuable natural or cultural resources and an untapped agritourism potential; instead, they were oriented at regions dominated by semi-subsistence or family farms. For a large part of farmers, the new form of support is about to become a source of additional incomes.

## Introduction

Agritourism is a form of tourism which emerged in the last century to become an increasingly important sector of the tourism industry around the world. The changing lifestyles and tourist behaviors and preferences, combined with concern for the cultural heritage, natural environment and sustainable development, led to the establishment of agritourism as a separate sector. The literature addresses two essential trends in agritourism. The first one is related to small family farms dispersed over rural areas which are used as accommodation facilities where hosts themselves provide services to guests [1, 2, 3, 4, 5, 6]. The second trend suggests that agritourism farms gradually enter, and become highly competitive in, the tourism market [7, 8, 9, 10, 11, 12]. As emphasized by many researchers (the list of individual items in Table 1), the common characteristic of these trends is that agritourism activities contribute to preservation of regional (and family) heritage and represent an additional non-farming income for the farmers. In this context, it should be noted that the domination of valuable natural habitats is usually not conducive to intensive agricultural production. As a form of tourism offered in a family farm, agritourism is an economic activity which, as such, has a broad impact on socioeconomic relations and rural landscape [13]. As repeatedly emphasized by the European Union, the farmers’ activity may encourage investments and attract more tourists [12]. However, in order for this to happen, programs and measures put in the context of sustainable development need to be developed and implemented. Indeed, in order for this form of economic activity to develop, it is usually advisable to preserve the high quality of the natural environment and cultural values. Generally, this requires the involvement of public funds. Therefore, this concept clearly refers to economic, social and environmental dimensions of sustainable development.

**Table 1:** Selected definitions of agritourism.

	Terminology used	Definition	Author
International	Agritourism	‘‘any practice developed on a working farm with the purpose of attracting visitors”	[29]
		‘‘a specific type of rural tourism in which the hosting house must be integrated into an agricultural estate, inhabited by the proprietor, allowing visitors to take part in agricultural or complementary activities on the property”	[30]
		‘‘activities of hospitality performed by agricultural entrepreneurs and their family members that must remain connected and complementary to farming activities”	[39]
	Agrotourism	‘‘tourism activities which are undertaken in non-urban regions by individuals whose main employment is in the primary or secondary sector of the economy”	[40]
		‘‘tourist activities of small-scale, family or co-operative in origin, being developed in rural areas by people employed in agriculture”	[41]
		‘‘provision of touristic opportunities on working farms”	[42]
	Farm Tourism	‘‘activities and services offered to commercial clients in a working farm environment for participation, observation or education”	[43]
		‘‘a part of rural tourism, the location of the accommodation on a part-time or full-time farm being the distinguishing criterion.”	[44]
	Vacation Farms	“incorporate both a working farm environment and a commercial tourism component”	[45]
in Poland	Agritourism	“involves staying in a rural household and includes various forms of leisure activity and tourist services delivered within a farm”	[32]
		“refers to supply-side operators representing the interests of farms who offer tourist services”	[46]
		“tourism which includes any and all manifestations of tourist services outside urban areas; tourism related to agriculture (agritourism) which does not restrict the farmer’s services to accommodation and catering; and tourist stays in a farm (farm tourism) whose agricultural functions are overshadowed by tourist services”	[47]
		“ensuring attractive leisure and improved health (especially physical fitness), getting to know the regional culture, rural living and working, and meeting new people”	[48]

Source: own compilation based on the referred literature

One of their tasks should be to highlight the role and importance of agritourism activities. In Poland, agritourism is equated with tourism activities run by farms whereas other European Union countries consider it to be a part of agriculture. Over the recent years, Polish agritourism has made considerable progress, and therefore became a topic to be addressed in order to identify practical business needs and to develop the relevant scientific analysis tools.

The purpose of this paper is to identify the development aspects of Polish agritourism with particular emphasis on natural and cultural attractiveness. To demonstrate the relationship between agritourism activities of Polish farms and the cultural and natural attractiveness, the Hellwig’s synthetic development indicator was used. While this analysis addresses specifically the Polish realities, the research method employed is of a more general nature. Despite the theoretical ambiguities of its definition, agritourism as a practical business is based on similar principles in different countries. It always involves rural areas and farms, especially in locations which boast numerous natural and man-made attractions.

## The concepts of agritourism and rural tourism

Agritourism primarily consists in organizing a stay for tourists (guests) in the farm, with optional catering, purchase (and participation in the production) of agricultural goods, and environmental leisure services [14, 15, 16, 17, 18, 19, 20, 21, 22]. The essence of agritourism is a part of multifunctional sustainable development of rural areas. According to [23, 24, 25, 26] “agritourism is a form of leisure activities which take place in agriculturally-oriented rural areas, and are based on accommodation facilities and recreation related to a farm or equivalent holding and its (natural, production and service) environment.” The German Federal Ministry for Food, Agriculture and Consumer Protection indicates that agritourism addresses the important aspect of rural employment. Combining tourism with agriculture is believed to be a diversification strategy [27].

Agritourism is a complex phenomenon, as reflected both in economic practice and in the relevant literature (Table 1). It involves economic, social and cultural aspects, and is a major driver of regional and local development. As a scientific field, it attracts interest from numerous researchers [28,29,30,31,32,33,34]. To understand the importance of agritourism, its essential features must be presented [28,35,36,37]. According to [38], that type of tourism must be consistent with several characteristics of a tourist destination. First, in a purely administrative sense, it should be restricted to agricultural areas rather than broadly defined rural areas. Second, a close relationship should exist between the host and his/her guests, which consists in using residential and farm buildings to offer accommodation services. The third element is to enable an active recreation based on farming activities. This includes cases where the guests take care of the animals or assist in crop production.

In Poland, many researchers [32, 33, 49,50, 51,52, 53, 54] present agritourism as a form of rural tourism because it extends over all forms of tourism related to farming activities and/or agricultural premises. Polish researchers believe that agritourism is related solely to activities performed on a farm whose owners organize a stay for their guests in where they live and work. According to [33, 55, 56], agritourism should be considered from the perspective of the customer (tourist) and service provider (farm owner). Hence, it can be defined as a specific production and service micro-enterprise whose activity consists in developing the best possible tourist products which is a form of leisure on a farm. Crop and livestock production are among the greatest attractions for tourists.

Agritourism may involve three key areas. The first is working on a farm; the second are the activities available to tourists on and outside the farm; the third means the experience lived by the tourists when staying on the farm [28]. Currently, a discussion is ongoing on how inclusive is the term ‘agritourism’ and how should its definition be developed [35, 36, 37]. However, the contact between the tourist and the farm, whether active or passive, has undoubtedly an important impact on his/her impressions, emotions and experience [57]. As emphasized in international literature, the essence of agritourism may be perceived in several dimensions. The first involves the farmers themselves and means that the farmer conducts an economic activity which consists in accommodating tourists (guests) in a household to earn non-agricultural income and create jobs for the farmer and his/her household members. The second dimension highlights the role of the guests, and means a specific way of traveling and enjoying leisure in a rural environment. The third one, in turn, is a reference to local development issues, and involves managing rural areas in line with the multipurpose model [58]. The concept of sustainable multifunctional development of rural areas is primarily about encouraging various business opportunities while addressing environmental concerns [59, 60, 61].

Obviously, the definitions of agritourism, as presented above, do not exhaust the complete list which may be found in the extensive literature on the subject. The European law fails to provide an unequivocal definition of agritourism. The member states were given the freedom to conceptualize that term [62]. In Poland, the applicable legal standards do not include the term “agritourism.” However, a number of legal acts exist which govern various aspects related to the delivery of tourist services. In turn, the European law takes “rural tourism” into account as a term extending over any manifestations of tourist activity in rural areas.

Agritourism does not only mean accommodation, catering and active leisure, but also getting to know the rural culture, customs, rituals and folklore. Currently, a number of controversies have arisen around agritourism which stir interest among the scientific community. This is because many terms, definitions, features and expressions are somehow similar and have overlapping meanings. Many authors describe different kinds and forms of tourism and farms from their own perspective, resulting in certain terminological ambiguities.

This is reflected in the relevant literature: for instance, the term ‘agritourism’ is used interchangeably with ‘rural tourism’ [29, 46, 57, 63, 64, 65]. However, it has to be clearly stated that agritourism is not a synonym for rural tourism. Instead, it is a more detailed area of rural tourism as a broader concept (Table 2).

**Table 2:** Selected definitions of rural tourism.

	Definition	Author
International	It is with village community as the activity place and with the unique production patterns, lifestyle and pastoral scenery of village as the object.	[66]
	A tourism activity with rural landscape as tourism attractions in rural areas.	[67]
	A small-scale, discrete tourism activity with sightseeing, vacation and leisure nature, based on various types of villages, with rural culture, rural life and rural pastoral scenery as a tourist attraction.	[68]
	Rural Tourism encompasses a huge range of activities, natural or manmade attractions, amenities and facilities, transportation, marketing and information system.	[69]
	Rural tourism can be defined as the country experience‘ which encompasses a wide range of attractions and activities that take place in agricultural or non-urban areas. Its essential characteristics include wide-open spaces, low levels of tourism development, and opportunities for visitors to directly experience agricultural and/or natural environments	[64]
	Rural tourism connects tourism products. Rural tourism connects areas of rural leisure activities. Therefore the rural tourism, based on the rural circumstances, is a type of tourism which can be combined with the elements of cultural and active tourism (e. g.: horse riding and hiking).	[70]
	Rural tourism is defined as tourism where nature or the rural location are the main attractions	[71]
	Rural tourism is tourism which takes place in the countryside.	[72]
In Poland	the entire tourism economy in rural areas	[55]
	any forms of tourism practiced outside urban areas, including eco-tourism and agritourism	[73]
	the entire tourism economy in rural areas in their functional meaning (referred to as “true rural areas”) which positively affects their multi-purpose development	[74]
	The potential of the natural environment, especially including protected areas	[75]

Source: own compilation based on the referred literature

In Poland, rural tourism can be defined as tourism activities in rural areas. According to international literature, rural tourism was a new kind of tourism which emerged in late 1800s and experienced tremendous growth after World War 2.

Rural tourism is similar in many, though not all, aspects in all countries around the world [37]. As indicated by [71], the United States witness the development of rural tourism because of several factors. First, there is growing interest in tradition, cultural heritage, authenticity and rural living. Second, people are more and more willing to enjoy a short (weekend) stay in rural areas. Third, the population of large cities becomes increasingly aware of health issues. Rural areas become attractive not only due to their agricultural nature but also because they address numerous needs, such as fresh air or active leisure. In the European Union, rural tourism is often equated with a segment of the economy that pools together private providers of accommodation services who offer both accommodation and various attractions. Rural tourism creates the identity of the location concerned by encouraging the guests to visit it and enjoy the numerous attractions. Note however that the services differ in quality standards across regions and countries [72].

In this paper, agritourism is defined as an additional activity of farms which is focused on hosting guests and providing them with accommodation and other attractions, if any.

## Development of Polish agritourism after Poland’s accession to the EU

In European Union countries and in Poland, the development of agritourism started in the 1990s, although the first concepts of using rural areas for leisure purposes can be traced back to the 19^th^ century [76, 77]. Agritourism takes various forms depending on natural conditions, tourism development stage etc. [78,79].

The 1990s witnessed a rapid development of agritourism around the world, especially in Europe. The five European countries with the largest number of agritourism facilities are Austria, Germany, UK, France and Ireland; all of them have a well organized agritourism sector. In Poland, agritourism is considered to be among the key rural tourism activities, and its development is based on using rural farms as accommodation facilities. In recent years, Europe and in Poland has witnessed the increasing importance of multifunctional farms as an alternative strategy to growth in the context of structural change in agriculture and adjustments to the European agricultural policy. Poland enjoys favorable conditions for agricultural development, especially in regions characterized by extremely low industrialization and urbanization levels, small shares of non-agricultural employment, high unemployment figures and small incomes earned by owners of vacant residential properties. The monitoring of rural areas [80] indicates the basic reasons for rural unemployment and poverty, such as the demise of state-owned farms and industrial plants in small towns in early 1990s, and the fragmentation of agricultural land. Many locations affected by these developments are also highly attractive to tourists, which provides a development opportunity for poor regions. In agritourism, as an activity related not only to rural areas but also to agriculture, the situation of small farms plays a particularly important role. Their small production potential makes them unable to ensure full employment of all family members. Moreover, because of the particularities of the Polish social insurance system, farm users cannot be registered as unemployed and are ineligible for unemployment benefits. In Poland, the interest in agritourism is driven by a series of objective processes which reflect the economic and social interests of three population groups: farmers (who access an additional source of non-agricultural income); urban residents (who satisfy their need for low-cost leisure in a calm and healthy environment); and rural communities (who expand their local government budget with additional local taxes from aritourism). The Polish literature provides many analyses and assessments of agritourism. However, focus should be placed on prerequisites for the development of that specific area of tourism. In Poland, the number of agritourism farms largely varies from one region to another (Table 3 based on [81]).

**Table 3:** Number of agritourism farms in Poland.

Region	Facilities	Number of beds
Dolnośląskie	616	7137
Kujawsko-Pomorskie	234	2836
Lubelskie	456	3936
Lubuskie	108	1143
Łódzkie	165	1719
Małopolskie	1327	16072
Mazowieckie	364	3610
Opolskie	119	1211
Podkarpackie	985	8516
Podlaskie	625	5803
Pomorskie	672	7595
Śląskie	404	5172
Świętokrzyskie	313	2855
Warmińsko-Mazurskie	801	7696
Wielkopolskie	438	4952
Zachodniopomorskie	389	4329
Total	8016	84582

Initially, the strongest development of Polish agritourism was observed in northern and southern regions. The key reasons behind the different spatial development pattern are the geographic location, historical background, natural variation across the country and economic disparities between regions [82]. The interest in running an agritourism business became particularly noticeable after Poland’s accession to the European Union. The broad range of measures under the 2004–2006 and 2007–2013 Rural Development Programs (RDP) enabled agritourism to embark on a sound development path and be promoted among potential customers. According to reports and papers of the largest Polish paying agency (the Agency for Restructuring and Modernization of Agriculture), over PLN 317 million was allocated to agritourism farms under the 2007–2013 RDP [80]. The development of rural tourism is not only of key importance to farmers; it also improves the standard of living for the rural population as it brings benefits from social development [83, 84, 85, 86, 87, 88, 89, 90]. It also conveys values related to the development of environmental knowledge, and moulds attitudes towards cultural, artistic and natural resources of the region concerned [91, 92, 93, 94].

## Cultural and natural attractiveness of Poland

Polish rural areas are an attractive place to live, work, relax and run a farming or non-farming business. In Poland, rural areas account for over 93% of the national territory which is inhabited by ca. 38% of the population [95]. They are characterized by a diversity of tourist products, are a beacon of culture and tradition, and a place where unique natural and landscape values are preserved. Agritourism businesses launched by farmers provide new opportunities regarding the use of rural areas which undoubtedly is in line with the principles of sustainable multifunctional development. A rapid development of non-agricultural activities is observed in areas with less favorable soil and climate conditions which are discouraging to agricultural producers. Usually, these areas offer favorable conditions for the development of the tourism sector. In Poland, areas characterized by a high potential for tourism development include mountainous regions (Carpathians, Świętokrzyskie Mountains, Sudetes), coastal (Baltic) regions and the great lakes (Suwałki lake district, Warmia-Masuria) [96]. Another finding is that agritourism dominates mainly in six regions, namely in Podkarpackie, Małopolskie, Pomorskie, Zachodniopomorskie, Warmińsko-Mazurskie and Podlaskie region [96]. Also, some isolated districts may be identified in Poland which demonstrate high natural values affecting the development of agritourism. The development of Polish agritourism also depends on many other factors: activity of the society and local government; natural and cultural assets; development of tourist facilities; and infrastructural conditions [61]. The landscape and its characteristics are among the core conditions for agritourism development. When visiting rural areas and agritourism farms, the tourists (guests) want first of all to enjoy what they miss in cities: calm, nature and landscape. What makes landscape a valuable tourism asset and encourages tourist activities are its water resources (rivers, lakes, creeks, swamps) and land features (forests, meadows, mountains, hills, beaches, plants, animals).

The main advantage of Polish agritourism is that the guests may enjoy an active stay, appreciate the rural landscape and contact the rural population to learn their culture, customs and ceremonies. This is because agritourism is based on locally available human and material resources. While it may be a driver of rural development, note that this is not true for all rural areas as only some of them offer adequate natural or cultural values. In Poland, there are districts with extraordinary opportunities for the development of agritourism. Note also that agritourism is undoubtedly a source of additional income for farmers and a factor affecting the development of rural landscape.

## Methodology

To demonstrate the relationship between agritourism activities of Polish farms and the cultural and natural attractiveness, the Hellwig’s synthetic development indicator was used. It essentially consists in calculating a synthetic indicator which specifies the size of a specific complex phenomenon based on several diagnostic features. In this study, two synthetic indicators were defined for the following phenomena:

1. natural attractiveness,
2. cultural attractiveness.

Natural attractiveness is defined as the attributes of a specific location which result from biological or geographical (e.g. geological) processes rather than from human activity. Conversely, cultural attractiveness is of an anthropogenic nature and includes selected effects of human civilization. For a detailed list, see Tables 4 and 5. Both groups of characteristics were selected in line with the assumption that the objective of visiting agritourism farms is not only to stay at the farm itself but also to explore local attractions.

**Table 4:** Diagnostic features used to formulate the Hellwig’s synthetic indicator of natural attractiveness.

Diagnostic features	Type of characteristics	Data source
Share of legally protected areas (district area = 100)	stimulant	https://bdl.stat.gov.pl/BDL/start
Share of forests (district area = 100)	stimulant	https://bdl.stat.gov.pl/BDL/start
Share of water bodies (district area = 100)	stimulant	https://bdl.stat.gov.pl/BDL/start
Share of leisure areas (district area = 100)	stimulant	https://bdl.stat.gov.pl/BDL/start
Share of green areas (district area = 100)	stimulant	https://bdl.stat.gov.pl/BDL/start
Natural monuments / 100 km^2^	stimulant	https://bdl.stat.gov.pl/BDL/start

Source: own calculations, n = 315. Basic data avaliable in the “Supporting information” section in S1 Table

**Table 5:** Diagnostic features used to formulate the Hellwig’s synthetic indicator of cultural attractiveness.

Diagnostic features	Type of characteristics	Data source
Total number of facilities entered to the register of monuments / 100 km^2^	stimulant	https://www.danepubliczne.gov.pl/dataset/rejestr-zabytkow-nieruchomych
Tourists per 10,000 population	stimulant	https://bdl.stat.gov.pl/BDL/start
Total number of cultural and sport events per 10,000 population	stimulant	https://bdl.stat.gov.pl/BDL/start
Total number of participants to major events per 1,000 population	stimulant	https://bdl.stat.gov.pl/BDL/start

Source: own calculations, n = 315. Basic data avaliable in the “Supporting information” section in S2 Table

The process was split into the following stages [97]:

1. selecting the diagnostic features for the phenomena considered,
2. normalizing the values of diagnostic features,
3. determining the values of synthetic indicators,
4. determining the Hellwig’s synthetic development indicator,
5. determining the typology classes.

Diagnostic features (Tables 4 and 5) were selected based on the following factual and statistical criteria:

- availability of statistical data at district level (In European Union statistics, districts are NUTS 4 units. In Poland, a district is a local government unit at the 2^nd^ level of administrative division, comprising part of a region area [98]. Poland is composed of 314 districts and 66 urban districts (municipalities which also carry out the tasks of a district).
- high relevance,
- weak correlation with other characteristics of the same phenomenon (based on the analysis of diagonal entries of the inverse of the R correlation matrix).

All simple features used in the analysis are stimulating variables which means that high values are desired from the perspective of the complex aspect under consideration.

The normalization procedure consisted in converting the values of each indicators to ensure comparability by rescaling them and unifying their orders of magnitude. The following formulas were used for that purpose [97]:

for stimulants:

$$z_{ij}=\frac{x_{ij}-\underset{i}{\text{min}}\{x_{ij}\}}{\underset{i}{\text{max}}\{x_{ij}\}-\underset{i}{\text{min}}\{x_{ij}\}}$$ (1)

where:

x_ij_ (i = 1, 2, …, n; j = 1, 2, …m) is the value of diagnostic features *j* in district *i*.

The synthetic indicators of different phenomena were determined using the pattern-based method which consists in calculating the distance of an individual unit from the pattern. The distance is calculated as follows, based on the normalized values of characteristics under consideration:

$$q_{i}=\sqrt{\frac{\sum_{j=1}^{m}{\left(z_{ij}-z_{0j}\right)}^{2}}{m}}$$ (2)

where:

z_0j_ is the normalized value of indicator *j* of the pattern which is such that:

$${\text{z}}_{0\text{j}}=\text{max}\left\{{\text{z}}_{\text{ij}}\right\}$$ (3)

The Hellwig’s synthetic development indicators were calculated as follows:

$${\tilde{q}}_{i}=1-\frac{q_{i}}{q_{0}}$$ (4)

where:

$$\begin{array}{lll} q_{0}={\bar{q}}_{0}+2s_{0} & {\bar{q}}_{0}=\frac{\sum_{i=1}^{n}q_{i}}{n} & s_{0}=\sqrt{\frac{\sum_{i=1}^{n}{\left(q_{i}-{\bar{q}}_{0}\right)}^{2}}{n}} \end{array}$$

The classes that indicate the degree to which the objectives of different phenomena were attained are determined based on the Hellwig’s synthetic development indicators. The corresponding arithmetic average $\bar{q}$ and standard deviations (s_q_) were used for that purpose:

$$\begin{array}{ll} \text{class}\;1: & {\tilde{q}}_{i}\geq \bar{q}+{\text{s}}_{\text{q}}\\ \text{class}\;2: & \bar{q}+{\text{s}}_{\text{q}}>{\tilde{q}}_{i}\geq \bar{q}\\ \text{class}\;3: & \bar{q}>{\tilde{q}}_{i}\geq \bar{q}-{\text{s}}_{\text{q}}\\ \text{class}\;4: & {\tilde{q}}_{i}<\bar{q}-{\text{s}}_{\text{q}} \end{array}$$ (5)

A slightly different typology approach was used to group the districts by:

1. the amount of agritourism investments supported with 2007–2013 RDP funds (PLN/km^2^);
2. number of beds in agricultural accommodation facilities per 100 km^2^.

**Fig 1:**
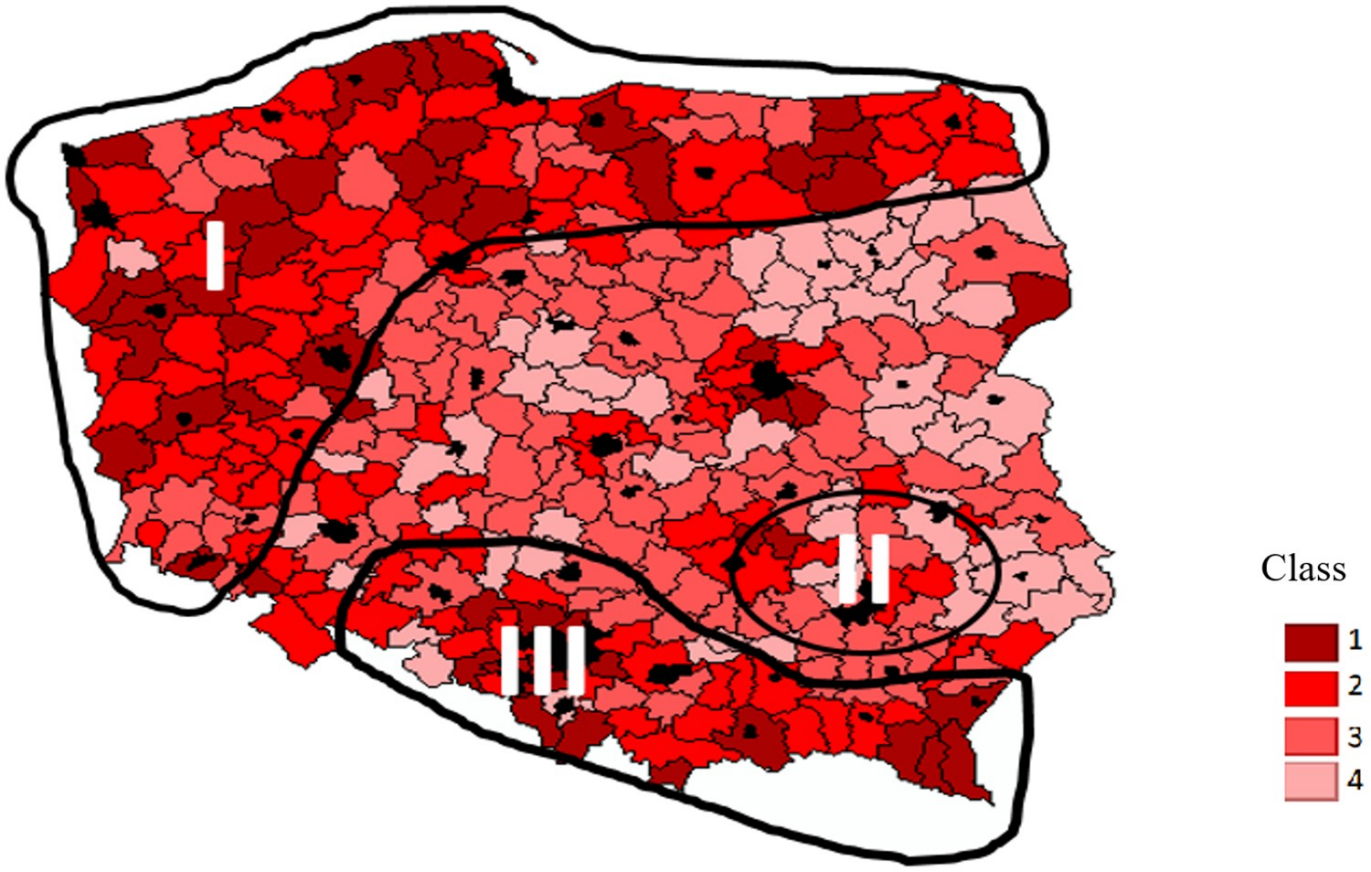
Natural attractiveness of Polish districts (based on the synthetic characteristic). Value of synthetic indicators: Class 1: 0 ≥ 0,060. Class 2: 0,060 ≥ 0,120. Class 3: 0,120 ≥ 0,179. Class 4: 0,179 ≥ 0,362. **Source: own study based on** Table 4 **data and** S1 Table.

**Fig 2:**
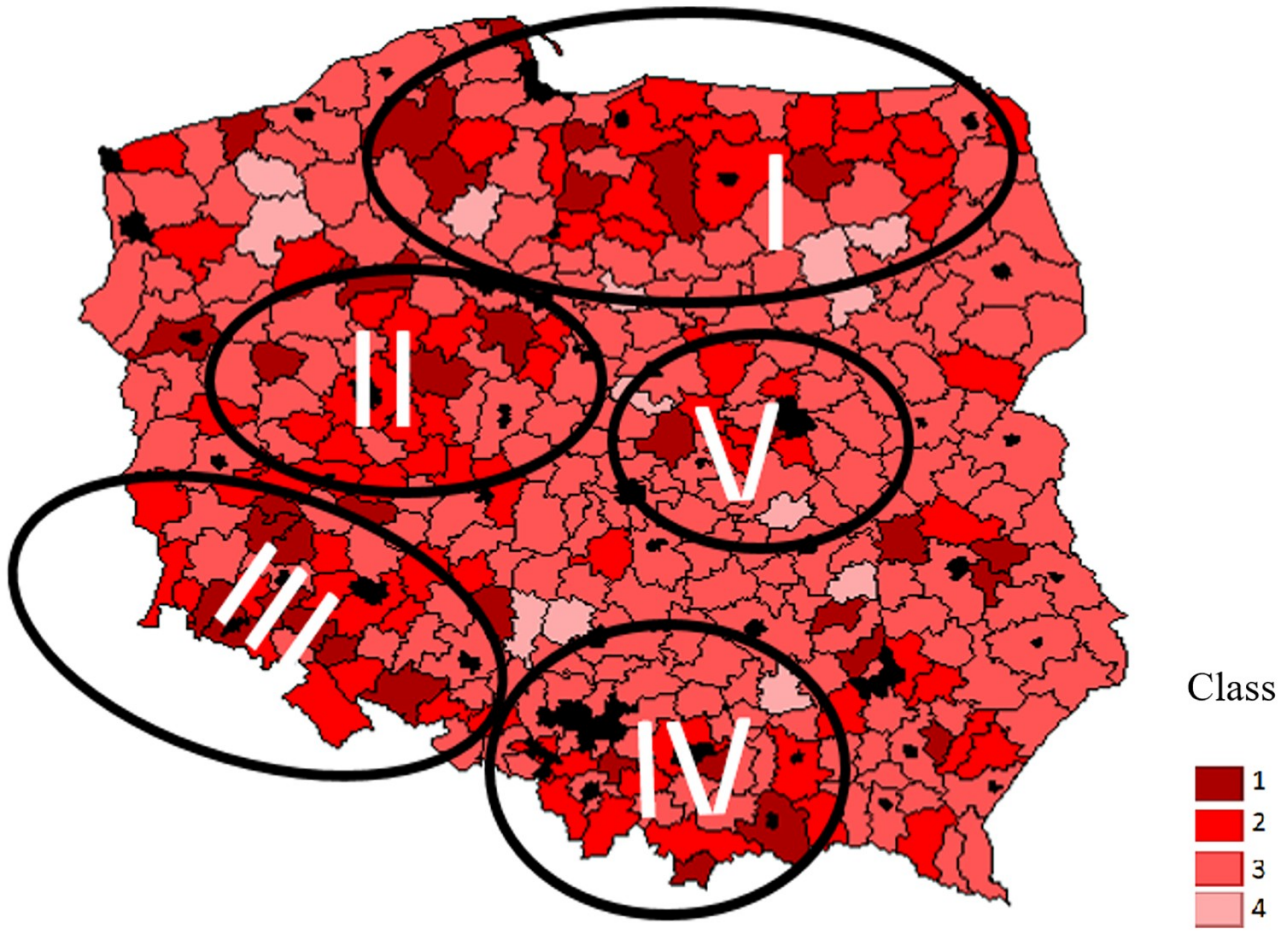
Cultural attractiveness of Polish districts (based on the synthetic characteristic). Value of synthetic indicators: Class 1: 0 ≥ 0,052. Class 2: 0,052 ≥ 0,104. Class 3: 0,104 ≥ 0,157. Class 4: 0,157 ≥ 0,364. Source: own study based on Table 5 data and S2 Table.

**Fig 3:**
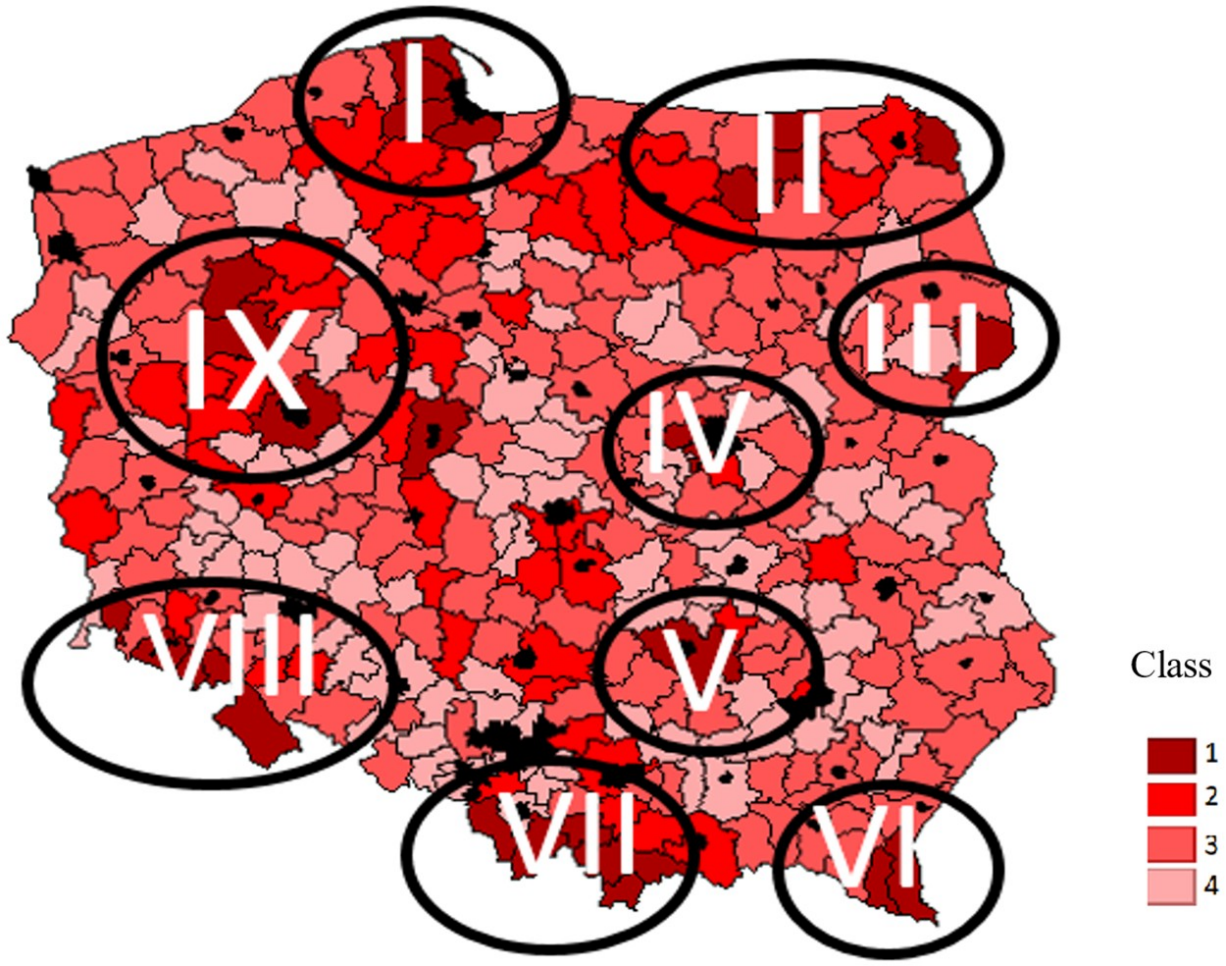
Number of beds in agritourism accommodation facilities per 100 km^2^. Number of beds / 100 km^2^. Class 1: 0 ≥ 0,1. Class 2: 0,1 ≥ 5. Class 3: 5 ≥ 10. Class 4: 10 ≥. Source: own calculations based on https://bdl.stat.gov.pl/ and S3 Table.

**Fig 4:**
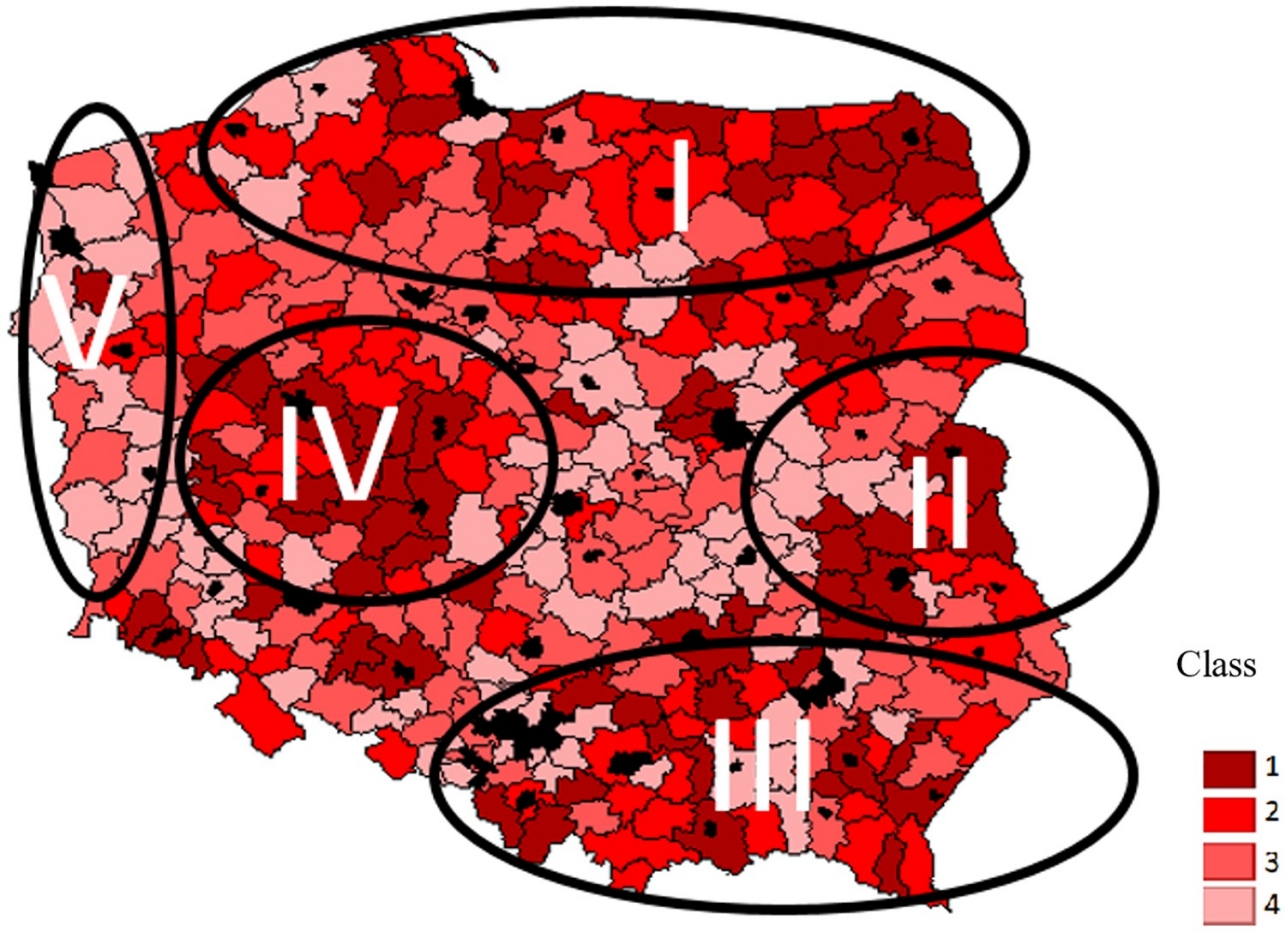
Amount of agritourism investments supported with EU funds (PLN/km^2^). Value of agritourism investments supported by EU (PLN/ km^2^). Class 1: 0 ≥ 650. Class 2: 650 ≥ 1300. Class 3: 1300 ≥ 2500. Class 4: 2500 ≥. Source: own study based on [27] and S3 Table.

Because one diagnostic feature exists in both cases, no synthetic indicators needs to be developed. However, the districts were grouped into four typology classes as per formula (5). The approach based on typology classes established for four sets of phenomena was used because the phenomena are different in nature. Also, it allowed to specify the strength of relationships between them, both with the use of the Pearson’s linear correlation formula (Table 6) and by visualizing them on a map (Figs 1–4). The maps show compact areas of the national territory with a high concentration of districts grouped in the first or second class with respect to different phenomena covered by the analysis.

**Table 6:** Pearson’s linear correlation coefficient for the phenomena under consideration.

Specification	Cultural attractiveness (size of the synthetic indicator)	Natural attractiveness (size of the synthetic indicator)	Eligible implementation costs (PLN/ km2)	Number of beds in agricultural accommodation facilities per 100 km2
Cultural attractiveness (size of the synthetic characteristic)	1.00	0.20 (p = 0.,001)	0.03 (p = 0.540)	0.19 (p = 0.000)
Natural attractiveness (size of the synthetic characteristic)	0.20 (p = 0.001)	1.00	-0.01 (p = 0.935)	0.30 (p = 0.000)
Eligible implementation costs (PLN/ km2)	0.03 (p = 0.540)	-0.01 (p = 0.935)	1.00	0.00 (p = 0.973)
Number of beds in agricultural accommodation facilities per 100 km2	0.19 (p = 0.000)	0.30 (p = 0.000)	0.00 (p = 0.973)	1.00

Source: own calculations, n = 315. Basic data avaliable in the “Supporting information” section in S3 Table

The correlation analysis used the Hellwig’s synthetic indicator of development for the natural and cultural attractiveness, respectively (variables V1 and V2 in S3 Table), the eligible costs of agritourism operations (variable V2, PLN/km^2^) and the number of beds in agritourism accommodation facilities (variable V3, beds/100 km^2^). All values were determined for the districts covered by this analysis. In this study, the natural and cultural attractiveness is a set of exogenous conditions for agritourism development; the number of beds in accommodation facilities is supposed to the reflect the previous activity of farms, whereas the amount of eligible costs of agritourism investments is considered to be a reflection of their current and future activities.

The method used in this study has its limits. First of all, it relies on variables retrieved from public statistics, and therefore it addresses quite large territorial units and makes no direct reference to local attractions (at town level) which, at least potentially, could be an important driver of agritourism. Furthermore, it fails to consider the psychological aspects behind the motivation of both suppliers and customers of agritourism services. This means that agritourism, a widespread phenomenon, requires in-depth research, including a direct investigation into agritourism operators.

## Justification of the scope of research

I The research focused on spatial diversity of agritourism activity in the context of natural and cultural attractiveness. The current number of agritourism farms and planned investments financed from EU funds has been taken into account It may be hypothesized that the decisions related to previous and planned activities were driven by slightly different motives. Supposedly, in the first case, a purely market-based approach was used which consists in leveraging the environmental advantages (primarily including the natural or cultural attractiveness) to maximize the economic effects. Having in mind the relatively short history of non-repayable EU funds in the Polish economy, it may be assumed that most previous agritourism projects were financed either with the farmers’ own funds or with bank loans (commercial or preferential loans). That financing method needs to be based on a strict economic calculation; the investor must establish a business plan which is credible both to himself/herself and to the bank. In practice, this means taking account of a series of exogenous and endogenous conditions, especially including the abovementioned natural and cultural assets of the place where the agritourism business is run. Both the providers of capital (mainly banks) and investors are required to apply strict economic criteria because all of them put their capital at risk. The situation gets different if EU funds are accessed (under the 2007–2013 Rural Development Program). Seemingly, the requirements should be the same, as the same kind of activity is involved. However, non-refundable support (even if the beneficiaries are required to make their own contribution) may perturb the economic calculation, making certain operators likely to start a business in locations where market success cannot be guaranteed. In this case, the risk of failure will be partially borne by the Union’s taxpayers rather than by the investor.

## Results and discussion

Agritourism is an offering of leisure activities underpinned by natural or, where possible, cultural assets. The former are more important because the rural location of agritourism operators directly reflects the dominant role of such characteristics as nature, quietness and remoteness from urban hustle and bustle. This opinion is shared by [99], [100] and [101], who emphasize that agritourism plays an important role as it ensures contact between humans and nature. [100] indicates that Italian tourists who stay in agritourism farms appreciate the natural values [usually, vineyards] surrounding the farm while also trying local products. This is also what the customers expect. The cultural values are largely related to the urban environment, and therefore also refer to other forms of tourism. Nevertheless, some attractions related to human activity are located in rural areas or in nearby small towns. Therefore, when assessing the development drivers of agritourism, they also must be taken into consideration. As regards the development factors of Romanian agritourism, [102] focuses on aspects such as the picturesque beauty and richness of unaltered nature, hospitality of the owners and the richness of Romanian culture. As emphasized by American scientists, staying at a farm continues to be popular in the US and in many European countries, and the attractions located in agritourism facilities provide a great incentive for engaging in a broad range of tourist activities. Also, American scientists claim that the factors of decisive importance for the development of agriculture cannot be clearly identified because in addition to local natural resources, there also other important aspects related, for instance, to education or cultural events held in rural areas [103]. Therefore, the characteristics of an agritourism business may be expected to be highly convergent with the presence of natural and cultural assets (where available). This becomes even more obvious when considering the fact that valuable natural habitats are usually not conducive to intense, economically viable agricultural production, and therefore expanding the farming activities with agritourism seems to be a reasonable approach. A similar view is expressed by [80] and [104]. This is why it is important to determine the strength of relationships between natural and cultural assets, on one side, and agritourism activities, on the other.

However, as shown by research at district level in Poland (Table 6), the relationship is quite weak or, in some cases, does not exist at all. The greatest correlation was found between natural attractiveness (defined with a synthetic indicator) and the number of beds in agricultural accommodation facilities per 100 km^2^. However, it still was quite weak (0.30) which means that either some of the existing businesses were not established in the nature-attractive areasor that potential agri-tourists look for other advantages than those recorded in general official statistics. It might also suggest that some locations with valuable natural assets are not necessarily attractive to tourists or that their potential is not yet fully tapped. In turn, weaker correlation (0.19) exists between the number of beds in agritourism accommodation facilities and cultural attractiveness; it is easier to understand as this activity has more to do with nature tourism. Nevertheless, despite being relatively low, these are the highest values of all coefficients considered in this study. The above largely confirms that the previously applicable (more restrictive) forms of financing make the applicants more likely (or sometimes even force them) to take the location factor into consideration in their agritourism development plans. This is particularly evidenced by the nearly total lack of relationships between cultural and natural attractiveness, on one side, and the amount of costs of agritourism operations financed under the RDP (0.03 and 0.01, respectively), on the other. However, considering the complexity of economic developments and the multitude of motives behind individual business decisions, the hypothesis that the non-refundable nature of EU aid often distorts the economic calculations for the project (resulting in the risk being shared by taxpayers) should be considered as confirmed, at least partially. That opinion is also supported by the absence of relationships between the number of agritourism accommodation facilities in particular provinces and the amount of eligible operational costs (0.0). Another essential conclusion is that in Poland, agritourism is a non-agricultural activity usually carried out by farms with small amounts of agricultural land. Also, the Union funds they accessed were mainly allocated to investments in the outward extension, upward extension, repair and equipment of the agricultural holding’s residential and farm buildings intended for agritourism activities [105]. Meanwhile, many Polish researchers emphasize that non-refundable funds (disbursed as a Union grant) allocated to the development of agritourism should be aligned with the diverse levels of rural development, including the tourist potential and diverse barriers to development. Union funds should support projects that combine agriculture, rural tourism and natural and cultural heritage [84, 106]. As regards the risk transferred from private capital suppliers to public institutions, [107] notes that non-refundable funds are a justified way of supporting agritourism projects because of the higher operational risk which makes the banks reluctant to provide financing.

Assuming that previous locations were chosen on more reasonable grounds (which is confirmed by relatively high correlation levels), two options may be considered for projects supported with public aid. The first one is that the decisions were unreasonable in terms of how landscape values are used; the second option is that deliberate decisions were made to establish new facilities in less attractive locations which, however, had not been previously used for agritourism purposes. In this context, note that—at least to a certain extent—agritourism includes cases where residents of big cities opt for a short-term (weekend) getaway; for them, the key factor is the distance to be traveled rather than the numerous attractions. [108] and [109] emphasize that big city dwellers prefer short trips to nearby destinations. This can be illustrated by the example of the inhabitants of Warsaw who visit the Kampinos National Part located close to the city.

In the second case, public aid would be justified due to potentially smaller economic effects of business operations conducted in these locations. Obviously, this does not invalidate the previous opinion that risk is shared between the investor and the taxpayer. Referring to the situation in Slovenia, [110] notes that EU aid is not enough to enable the development of agritourism farms because the basic barrier they face is their small size and insufficient education of the owners. Another view is presented by [111] who, during a survey conducted in Poland, discovered that owners of agritourism farms are more entrepreneurial, innovative and active in accessing aid measures than owners of other small and medium enterprises.

Natural attractiveness, determined based on the synthetic characteristic, varies quite strongly across the country (Fig 1). Three essential areas may be identified with a dominant share of districts grouped in the 1^st^ and 2^nd^ typology classes. The first area (Area I in Fig 1) includes the coastal territories and postglacial lakes in the northern part of the country (Warmińsko-Mazurskie, Pomorskie and Zachodniopomorskie region and the northern part of the Podlaskie region) and districts with considerable forest coverage in the western part of the country (Lubuskie region and the western part of Wielkopolskie region). A similar view is also presented by [112] and [18]. Also, this area is largely covered by meadows, especially in the north-eastern part (the Warmia and Masuria region). Area II consists of low (Świętokrzyskie region) mountains whereas area III is the south of Poland with two mountainous ranges: Carpathians in the west and Sudetes in the east. The central and eastern part of the country is the least attractive in terms of natural assets. In their study, [113] also agreed that the central part of the country is the least attractive in terms of natural values.

Cultural attractiveness, determined based on the relevant synthetic indicator, is also highly variable (Fig 2). In some areas, high levels of cultural attractiveness go hand in hand with high natural attractiveness, but the correlation between the two indicators is relatively weak (0.20, see Table 6). However, sites of particular cultural interest are much less numerous and mostly located away from each other. The first compact region (Fig 2, area I) is the area of Polish and Teutonic castles and historic towns located in north-eastern Poland. Region II is mostly located in Greater Poland, the cradle of the Polish state and one of the country’s wealthiest regions which therefore is able to offer many moderns attractions, such as cultural and sports events. A similar situation takes place in region III, located in Lower Silesia, which also is among the wealthiest parts of Poland and is home to many old German castles (Until the end of World War II, it was part of the German territory). Region IV, Lesser Poland, is the location of the most important historical capital, Krakow, and is also home to numerous medieval castles and historic towns. Area V is Mazovia, the region where today’s capital, Warsaw, is located. It follows from the above that areas of particular cultural interest are mostly located around the country’s core historical sites, especially including the historical and modern-day capitals. Therefore, these are mainly urban areas which may be one of the reasons for relatively low levels of correlation with agritourism characteristics. Obviously, this pattern is not unique to Poland; cultural activity is largely an urban matter all around the world. However, note that enjoying urban attractions located within a relatively short distance from agritourism farms may be an additional value for customers who travel by their own car.

The map of previous agritourism activities, presented as the number of beds in accommodation facilities per 100 km^2^, only partially coincides with naturally and culturally attractive areas, and therefore the two characteristics are poorly correlated. This may be due to several reasons. Firstly, not all valuable natural areas have equally valuable tourism assets. Secondly, because of limited demand for agritourism services, the holdings are mostly located in areas of special importance. This is why the highest concentration of agritourism sites is observed in the coastal region (I), the Masurian Great Lakes district (II), Białowieża Forest (III), near big cities (IV and IX) and in mountain regions: Świętokrzyskie Mountains (V), Bieszczady (VI), Tatra and Pieniny (VII) and Carpathians (VIII). This means that while previous activities were largely based on an advantageous location, Poland offers more attractive cultural and natural venues. However, it may be expected that their future use for agritourism purposes will greatly depend not only on the availability of capital needed to build new accommodation facilities but mostly on the growing demand and marketing activities of the local government.

The geographic distribution of agritourism investments co-financed with EU funds is slightly different (Fig 4). First of all, the areas dominated by districts grouped in upper typology classes are much more numerous than in the case of the corresponding index calculated for the number of beds in accommodation facilities (Fig 3). Furthermore, they only partially coincide with areas characterized by the presence of valuable natural or cultural resources (Figs 1 and 2). Area I in Fig 4 includes only a part of the Great Masurian Lakes region. Most of its southern part is northern Mazovia which, according to the criteria used in this study, does not offer any natural or cultural attractions. However, it is dominated by small farms which may consider agritourism as a development opportunity, especially if the essential investments are co-financed with public funds. Another important factor is the closeness of Warsaw, the largest Polish city, which is home to many potential customers of agritourism farms. A similar situation takes place in area II which includes the Lubelskie region, yet another region dominated by semi subsistence farms. Region III, composed of districts located in Podkarpackie and Małopolskie regions (i.e. mountain areas), demonstrates considerable consistency with the location of natural and cultural assets and with the number of beds in agritourism accommodation facilities. These areas are also dominated by small farms, usually characterized by poor soils, which is typical of upland regions. Slightly different conditions prevail in region IV which mostly includes eastern parts of Greater Poland. This is all the more interesting since the western part of that region, typified by a very high proportion of forests and lakes, demonstrates greater natural values. The possible explanation is that small and medium family farms play an important role in the eastern part of the Wielkopolskie region. Just as in regions I and II, they look for an opportunity to supplement their incomes. The reason why they did not do so earlier was the excessive risk which now is transferred to European Union institutions (and, ultimately, to Union’s taxpayers). Area V is particularly interesting in the context of relationships between the structure of farms, natural attractiveness and agritourism activities. It includes the western part of Poland which offers valuable natural assets due to a significant share of wooded areas. Before World War II, it was part of the German territory; in the post-war era, communist authorities established large state-owned agricultural holdings in this region. After the economic reform in the 1990s, these became the basis for the creation of large private farms (mainly including family holdings). Therefore, despite its natural attractiveness and proximity to the German market (which became even more accessible after Poland joined the Schengen Zone), this region does not provide enough economic incentive to engage into additional agritourism projects because the levels of farm income are high.

## Conclusions

Agritourism, as a specific business which extends the scope of normal farming activities, is consistent with the concept of multifunctional rural development. Accordingly, rural areas should not only produce food but also offer space for leisure and non-farming business activities. Note that operators of agritourism farms follow the same general rationale as any other entrepreneur. First of all, they are committed to maximize their economic performance through an appropriate use of exogenous and endogenous conditions. In the case of agritourism, the cultural and natural attractiveness of a destination are an important exogenous factor. As shown by research, previous agritourism projects implemented in Poland took good account of the location factor. The highest concentration of beds offered by agritourism accommodation facilities was recorded in areas with particularly valuable natural assets. However, the agritourism potential of some slightly less attractive locations is not yet tapped. One possible reason is the financing method used in previous agritourism projects. The use of own capital and bank loans had to be backed up by strict economic calculations and marketing plans, including risk assessments. Things are different when it comes to current projects co-financed with Union public funds. The intensified efforts undertaken by the farmers to access Union financing were not focused on areas with valuable natural or cultural resources and an untapped agritourism potential; instead, they were oriented at regions dominated by semi subsistence or family farms. This means that, as regards at least a large part of beneficiaries, the new form of activity is supposed to become a source of additional income, which itself is consistent with the concept of entrepreneurship. However, they often fail to take account of valuable local assets (which is a key issue in any form of tourism); this may suggest that the economic and marketing analyses are not carried out or are not stringent enough. The underlying reason may be public aid granted on a non-refundable basis. Neither the funder (i.e. the European Union in a broad sense, represented by specialized paying agencies) nor the beneficiary do risk their own money; this may often distort the economic calculations, including risk assessments. Obviously, the above does not mean that public support for business is unfounded in principle. Especially in countries or regions with relatively low average incomes (which, on an EU-wide basis, include Poland), there is a considerable number of operators who are perfectly positioned to run an effective business but lack the necessary capital resources. Providing them with support (which is among the major aspects of the regional policy) is supposed to reduce the regional disparities. Supporting the development of agritourism, as a business usually run by small and medium family farms unable to grow using their own resources, perfectly fits that concept. However, it is necessary to redefine the assessment criteria for different projects co-financed with public funds in order to take better account of business and marketing aspects. This is because public paying agencies naturally pay more attention to formal requirements than to the assessment of economic viability and market risks.

This paper is not extensive enough to answer all the problems tackled. Therefore, it is necessary to carry out additional, more detailed research addressing three essential areas: the needs of potential customers of agritourism farms (the demand side); the offering of agritourism farms (the supply side), taking into account both exogenous factors (related to the farm itself) and exogenous factors (related to the farm’s location, especially including local natural and cultural attractions); and agritourism support programs (agricultural and regional policy). Particular emphasis needs to be placed on mutual compatibility of these three areas. This means checking whether the offering of agritourism farms meets customer expectations, and whether public support helps meeting customer expectations or is justified by any other reasons. The relevant research should be primarily carried out at local rather than at national level.

The findings of this study are also a basis for some recommendations, both for agritourism farm operators and for the authorities in charge of allocating public funds to this kind of activity. Farmers who either continue or intend to start an agritourism business should consider both the internal (farm-related) factors and the external ones, related to environmental attributes. This kind of activity is generally encouraged by the small size of the farm; by a production system based on self-supply; and by the resulting large amounts of untapped labor resources. As regards the destinations located in the neighborhood and beyond, research findings indirectly identify two types of attractive locations. The first are areas with specific natural or cultural values while the second is the vicinity of big cities. The recommendations for authorities in charge of supporting the development of agritourism (which may also be extrapolated to other forms of aid for undertakings) boil down to the requirement for a broader consideration of pure business factors (including analyzing the risk of financial non-viability of a project) with less focus on compliance with formal requirements.

## Supporting information

S1 Table Diagnostic features used to formulate the Hellwig’s synthetic indicator of natural attractiveness. Source: own calculations based on Polish Central Statistical Office data.
(PDF)

S2 Table Diagnostic features used to formulate the Hellwig’s synthetic indicator of cultural attractiveness. Source: own calculations based on Polish Central Statistical Office data.
(PDF)

S3 Table Variables for Pearson’s linear correlation coefficient for the phenomena under consideration. Source: own calculation based on data of Polish Central Statistical Office and Agency for Restructuring and Modernization of Agriculture.
(PDF)
