# Supplementary material for: Geographic differentiation of agritourism activities in Poland vs. cultural and natural attractiveness of destinations at district level
Source: PLoS One. 2019 Sep 20;14(9):e0222576. doi: 10.1371/journal.pone.0222576 (PMC6754142; doi:10.1371/journal.pone.0222576)
Supplement: S1 Table — Source: own calculations based on Polish Central Statistical Office data. (PDF) [file pone.0222576.s001.pdf]

S1 Table. Diagnostic features used to formulate the Hellwig's synthetic indicator of natural attractiveness

District	Share of legally protected areas (district area = 100)	Share of forests (district area = 100)	Share of water bodies (district area = 100)	Share of leisure areas (district area = 100)	Share of green areas (district area = 100)	Natural monuments / 100 km^2	Class
aleksandrowski	20,5	7,8	1,8	0,1	0,6	5,5	3
augustowski	64,1	47,2	3,5	0,1	0,1	3,6	2
bartoszycki	19,7	24,2	0,8	0,1	0,3	12,4	3
bełchatowski	28,0	29,9	0,7	0,1	0,4	9,9	3
będziński	2,2	20,7	2,3	1,2	2,6	13,2	1
bialski	9,0	27,2	0,6	0,0	0,1	9,1	4
białobrzeski	54,8	24,7	0,8	0,0	0,1	5,6	3
białogardzki	0,3	42,2	0,5	0,2	0,3	6,9	3
białostocki	34,1	40,1	1,4	0,1	0,1	4,8	3
bielski (śląskie region)	3,0	20,3	0,3	0,1	0,1	3,2	2
bielski (podlaskie region)	9,2	61,3	0,9	0,2	0,4	9,6	4
bieruńsko-lędziński	0,3	15,2	5,7	0,4	1,6	17,1	1
bieszczadzki	100,0	70,4	1,2	0,0	0,1	9,6	1
biłgorajski	10,9	39,3	0,4	0,0	0,1	2,7	4
bocheński	63,5	29,2	0,7	0,2	0,3	20,3	2
bolesławiecki	9,2	61,1	0,5	0,2	0,2	14,2	3
braniewski	30,3	26,5	6,0	0,1	0,2	16,6	2
brodnicki	55,2	21,5	3,1	0,1	0,1	10,1	2
brzeski (małopolskie region)	76,0	18,9	0,9	0,2	0,2	14,7	2
brzeski (opolskie region)	51,3	12,8	0,6	0,1	0,1	9,9	3
brzeziński	54,6	14,1	0,3	0,3	0,2	17,0	2
brzozowski	48,0	29,6	1,0	0,1	0,2	10,9	2
buski	96,5	11,1	0,7	0,1	0,3	3,1	3
bydgoski	41,1	41,7	2,7	0,2	0,2	12,8	2
bytowski	20,8	53,5	3,2	0,1	0,1	15,0	2
chełmiński	39,7	6,8	2,7	0,2	0,6	25,6	2
chełmski	39,8	18,6	0,4	0,0	0,2	4,1	3
chodzieski	30,1	36,2	1,2	0,1	0,2	12,3	2
chojnicki	58,8	52,7	4,7	0,1	0,3	16,6	1
choszczeński	54,6	39,6	2,8	0,1	0,2	9,8	2
chrzanowski	22,9	39,3	0,9	0,3	1,5	30,5	1
ciechanowski	37,5	16,9	0,3	0,1	0,3	10,5	3
cieszyński	33,2	38,9	1,2	0,4	0,7	24,5	1
czarnkowsko-trzcianecki	45,1	52,4	1,0	0,1	0,1	11,6	2
częstochowski	22,4	29,6	0,5	0,1	0,1	5,5	3
człuchowski	10,6	50,6	2,1	0,1	0,5	5,6	3
dąbrowski	11,0	11,4	1,3	0,1	0,1	2,5	4
dębicki	4,9	25,7	1,1	0,2	0,3	6,6	3
drawski	46,9	47,9	5,9	0,2	0,1	16,8	1
działdowski	38,2	29,5	1,5	0,1	0,2	11,7	3
dzierżoniowski	17,9	21,0	0,5	0,4	1,1	22,5	2
elbląski	41,0	19,6	9,5	0,1	0,1	42,4	1
ełcki	50,3	22,6	7,5	0,1	0,2	2,6	2
garwoliński	36,8	30,7	1,4	0,1	0,2	7,2	3
gdański	48,9	18,9	2,0	0,2	0,4	9,7	2
giżycki	69,1	26,7	13,4	0,2	0,2	12,8	1

gliwicki	11,0	34,0	1,8	0,2	0,7	9,5	2
głogowski	2,8	22,6	2,2	0,3	1,1	8,4	2
głubczycki	14,3	6,5	0,2	0,3	0,3	1,3	4
gnieźnieński	12,8	14,7	2,8	0,1	0,5	10,8	3
goleniowski	8,8	38,3	6,7	0,2	0,2	6,5	2
golubsko-dobrzyński	43,2	20,2	1,4	0,1	0,2	7,3	3
gołdapski	78,5	33,0	1,4	0,1	0,1	4,5	2
gorlicki	61,7	44,0	1,4	0,1	0,2	3,6	2
gorzowski	56,6	45,7	1,8	0,2	0,3	6,5	1
gostyniński	43,1	23,0	1,0	0,1	0,2	8,3	3
gostyński	18,6	14,5	0,3	0,3	0,4	14,1	3
górowski	70,5	29,4	1,0	0,1	0,1	11,4	2
grajewski	18,4	22,9	1,8	0,1	0,1	1,7	4
grodziski (mazowieckie region)	23,6	12,0	0,4	0,1	0,5	72,8	2
grodziski (wielkopolskie region)	13,5	6,8	0,2	0,1	0,3	41,6	3
grójecki	23,0	12,3	0,7	0,1	0,2	4,8	4
grudziądzki	33,4	14,7	2,5	0,1	0,1	26,1	2
gryficki	0,6	21,4	1,4	0,4	0,3	6,6	3
gryfiński	24,5	34,9	3,6	0,2	0,2	14,3	2
hajnowski	57,4	54,3	0,7	0,1	0,1	76,0	1
hrubieszowski	19,5	13,4	0,3	0,1	0,2	3,3	4
iławski	42,9	27,4	6,1	0,1	0,2	11,7	2
inowrocławski	10,5	10,5	2,8	0,2	0,6	20,2	3
janowski	63,0	41,3	0,4	0,1	0,1	15,5	2
jarociński	19,1	18,9	0,5	0,2	0,4	18,7	3
jarosławski	23,1	22,6	1,1	0,2	0,2	12,3	3
jasielski	37,3	37,6	1,1	0,1	0,3	4,3	2
jaworski	26,2	22,4	1,4	0,2	0,5	20,8	2
jeleniogórski	25,1	49,3	1,0	0,8	0,2	9,6	1
jędrzejowski	66,2	19,6	0,3	0,1	0,2	7,2	3
kaliski	24,5	20,4	0,5	0,1	0,2	4,7	4
kamiennogórski	16,0	38,5	1,0	0,3	0,2	8,3	3
kamieński	7,7	28,6	16,0	0,4	0,2	12,4	1
kartuski	49,2	31,0	5,2	0,1	0,1	11,0	2
kazimierski	19,1	2,9	0,4	0,1	0,1	3,8	4
kędzierzyńsko-kozielski	7,0	24,8	2,1	0,4	0,5	13,6	2
kępiński	15,8	20,3	0,2	0,1	0,5	5,4	4
kętrzyński	22,0	17,2	1,6	0,1	0,3	15,0	3
kielecki	91,0	35,2	0,6	0,2	0,1	4,5	2
kluczborski	33,6	30,7	0,3	0,3	0,4	6,5	2
kłobucki	7,7	29,7	0,5	0,1	0,1	4,4	4
kłodzki	37,5	44,2	0,5	0,3	0,4	11,1	2
kolbuszowski	49,3	36,6	0,4	0,1	0,1	6,2	3
kolneński	22,4	22,5	0,4	0,1	0,1	4,0	4
kolski	24,3	11,6	1,0	0,1	0,2	6,5	4
kołobrzeski	6,4	21,8	1,0	0,4	0,9	30,5	2
konecki	71,3	49,5	0,5	0,1	0,2	2,3	2
koniński	40,7	16,6	2,3	0,3	0,2	4,2	3
koszaliński	20,4	43,7	2,6	0,1	0,1	12,3	2
kościański	44,0	13,5	1,0	0,0	0,5	9,6	3
kościerski	54,9	45,3	5,0	0,2	0,1	7,1	1
kozienicki	11,9	32,8	4,0	0,1	0,2	11,2	3
krakowski	30,8	12,5	0,6	0,2	0,3	35,3	2

krapkowicki	16,1	24,8	1,5	0,7	0,8	8,6	2
krasnostawski	24,6	15,4	0,4	0,1	0,2	7,9	4
kraśnicki	23,7	20,3	0,7	0,1	0,3	6,8	3
krośnieński	26,5	62,3	2,0	0,2	0,2	6,6	2
krośnieński	37,1	87,2	2,9	0,3	0,3	9,3	2
krotoszyński	45,0	19,1	0,2	0,2	0,5	4,1	3
kutnowski	5,3	5,0	0,3	0,2	0,5	4,1	4
kwidzyński	31,0	23,6	3,7	0,1	0,5	13,9	2
legionowski	72,1	31,6	7,0	0,6	1,0	25,4	1
legnicki	12,0	16,0	1,1	0,2	0,3	20,4	3
leski	98,2	68,9	3,6	0,1	0,1	2,6	1
leszczyński	51,8	25,5	2,0	0,2	0,2	16,7	2
leżajski	44,0	31,6	1,1	0,2	0,3	2,1	3
lęborski	27,8	41,4	4,0	0,2	0,4	17,7	1
lidzbarski	24,2	28,3	1,5	0,1	0,1	6,2	3
limanowski	68,5	39,0	0,8	0,1	0,1	6,9	2
lipnowski	27,2	21,9	2,9	0,1	0,2	1,6	3
lipski	20,6	17,9	1,1	0,0	0,0	3,2	4
lubaczowski	47,8	48,2	0,4	0,1	0,1	14,2	2
lubański	2,3	24,4	1,0	0,5	0,5	36,2	2
lubartowski	25,0	19,7	1,2	0,1	0,2	3,5	3
lubelski	18,5	10,1	0,2	0,1	0,2	6,2	4
lubiński	2,1	32,3	1,0	0,4	0,7	11,2	2
lubliniecki	34,5	50,6	0,4	0,1	0,3	11,4	2
lwówecki	13,2	34,9	1,1	0,2	0,2	6,8	3
łańcucki	19,3	21,2	0,7	0,2	0,4	2,2	3
łaski	42,4	22,1	0,4	0,1	0,2	11,5	3
łęczycki	10,9	5,3	0,4	0,0	0,2	3,1	4
łęczyński	29,8	13,2	1,4	0,1	0,4	7,1	3
łobeski	2,8	32,5	2,4	0,4	0,3	6,3	3
łomżyński	25,9	22,8	0,9	0,0	0,0	4,0	4
łosicki	23,5	22,7	0,6	0,1	0,1	10,6	3
łowicki	20,5	10,2	0,4	0,1	0,1	3,5	4
łódzki wschodni	10,7	25,0	0,2	0,1	0,3	28,0	3
łukowski	16,0	22,4	0,1	0,1	0,1	4,6	4
makowski	1,0	25,8	0,9	0,0	0,1	3,7	4
malborski	8,3	2,3	2,4	0,3	0,7	15,2	3
miechowski	86,4	12,1	0,3	0,1	0,2	5,9	3
mielecki	13,2	24,9	1,2	0,2	0,6	3,7	3
międzychodzki	67,6	46,0	5,2	0,1	0,2	36,7	1
międzyrzecki	31,0	54,0	2,6	0,2	0,2	12,8	2
mikołowski	11,2	36,9	0,2	0,5	1,5	29,2	1
milicki	67,7	41,8	0,6	0,2	0,3	5,0	2
miński	29,6	21,8	0,3	0,1	0,2	17,4	3
mławski	50,2	20,1	0,3	0,1	0,1	6,4	3
mogileński	14,5	16,6	3,3	0,1	0,1	12,6	3
moniecki	31,6	20,7	0,9	0,0	0,1	1,1	4
mrągowski	60,6	32,3	12,3	0,1	0,2	7,6	1
myszkowski	35,7	24,1	1,3	0,1	0,2	4,8	3
myślenicki	11,6	35,7	2,5	0,2	0,2	15,2	3
myśliborski	43,6	42,9	3,1	0,2	0,4	11,1	1
nakielski	7,5	23,6	1,0	0,2	0,2	24,6	3
namysłowski	46,2	28,6	0,4	0,5	0,5	4,0	2
nidzicki	57,9	40,0	1,6	0,1	0,2	2,4	2
niżański	0,0	42,3	1,3	0,1	0,2	6,9	3

nowodworski (mazowieckie region)	60,8	26,5	3,3	0,1	0,2	10,1	2
nowodworski (pomorskie region)	62,9	27,4	3,4	0,1	0,3	10,4	2
nowomiejski	35,3	21,3	2,9	0,1	0,1	5,6	3
nowosądecki	81,6	44,1	2,2	0,1	0,6	10,1	1
nowosolski	21,4	40,2	1,4	0,2	0,4	13,2	2
nowotarski	88,5	37,5	2,1	0,1	0,2	5,8	2
nowotomyski	14,0	38,8	1,2	0,3	0,6	22,6	2
nyski	19,2	13,5	4,2	0,4	0,5	5,7	2
obornicki	14,2	32,1	1,3	0,2	0,3	24,8	2
olecki	40,0	27,0	3,9	0,1	0,1	5,1	2
oleski	10,3	36,2	0,3	0,1	0,1	4,5	4
oleśnicki	10,5	32,1	0,4	0,2	0,4	5,2	3
olkuski	33,0	37,0	0,3	0,2	0,5	12,5	2
olsztyński	53,9	39,1	4,6	0,1	0,1	4,5	2
oławski	0,1	20,2	1,5	0,3	0,6	4,2	3
opatowski	14,5	15,1	1,0	0,0	0,1	8,5	4
opoczyński	20,1	31,2	0,8	0,0	0,1	6,3	3
opolski (lubelskie region)	44,3	21,8	1,4	0,1	0,2	2,6	3
opolski (opolskie region)	23,4	11,5	0,7	0,1	0,1	1,4	4
ostrołęcki	0,4	31,4	0,4	0,0	0,0	3,8	4
ostrowiecki	73,4	31,2	0,5	0,2	0,6	4,7	2
ostrowski (mazowieckie region)	1,1	28,2	0,8	0,0	0,1	2,6	4
ostrowski (wielkopolskie region)	1,1	29,6	0,8	0,0	0,1	2,8	4
ostródzki	56,0	31,2	5,7	0,1	0,2	9,1	1
ostrzeszowski	84,2	35,5	0,4	0,1	0,1	9,6	2
oświęcimski	0,6	10,3	3,6	0,4	1,2	14,3	2
otwocki	78,0	30,5	2,8	0,1	0,4	22,4	1
pabianicki	18,2	26,6	0,4	0,2	0,9	12,8	2
pajęczański	5,8	25,1	0,7	0,1	0,1	9,6	4
parczewski	16,4	25,1	2,0	0,1	0,1	4,5	4
piaseczyński	52,5	19,5	2,5	0,1	0,9	24,3	1
pilski	42,5	29,4	1,5	0,2	0,3	12,0	2
pińczowski	98,6	18,4	0,5	0,1	0,2	4,7	3
piotrkowski	12,9	24,6	1,1	0,1	0,1	13,6	3
piski	58,0	50,5	10,4	0,1	0,1	7,2	1
pleszewski	9,9	19,6	0,4	0,4	0,8	20,2	2
płocki	33,7	17,0	3,6	0,1	0,1	9,1	3
płoński	35,7	14,3	0,8	0,0	0,2	9,1	3
poddębicki	24,2	15,1	1,7	0,1	0,2	8,3	3
policki	3,3	35,4	19,8	0,3	0,4	4,4	1
polkowicki	26,7	37,1	0,3	0,2	0,6	4,0	2
poznański	25,1	23,0	1,8	0,3	0,7	27,3	1
proszowicki	15,9	1,5	0,8	0,1	0,2	5,3	4
prudnicki	8,3	11,8	0,4	0,3	0,3	3,1	4
pruszkowski	37,3	11,3	0,5	0,4	2,2	47,6	1
przasnyski	3,2	30,9	0,3	0,0	0,1	5,7	4
przemyski	70,7	40,3	1,4	0,2	0,1	16,6	1
przeworski	45,0	24,4	0,8	0,2	0,3	20,9	2
przysuski	39,9	32,4	0,8	0,0	0,1	4,6	3

pszczyński	2,2	29,0	8,5	0,4	1,2	7,0	1
pucki	45,4	31,5	0,3	0,3	0,4	20,1	2
puławski	34,1	25,0	2,3	0,2	0,6	7,4	2
pułtuski	16,9	19,4	2,0	0,0	0,1	5,4	4
pyrzycki	0,1	6,6	5,0	0,3	0,3	4,1	4
raciborski	36,1	25,5	1,3	0,6	0,7	9,0	1
radomski	21,7	27,0	0,4	0,1	0,2	14,0	3
radomszczański	19,4	31,7	0,3	0,1	0,2	19,9	3
radziejowski	12,0	5,0	1,9	0,1	0,2	3,6	4
radzyński	0,9	21,4	0,5	0,1	0,1	5,1	4
rawicki	1,7	15,4	0,5	0,3	0,3	9,2	4
rawski	26,1	12,4	0,2	0,1	0,4	18,6	3
ropczycko-sędziszowski	33,0	25,3	0,5	0,2	0,2	6,6	3
rybnicki	56,7	33,5	0,3	0,3	0,6	8,5	1
rycki	11,8	21,9	1,3	0,1	0,3	11,2	3
rypiński	15,4	20,0	2,0	0,1	0,2	10,4	3
rzeszowski	39,5	24,3	0,6	0,2	0,2	7,5	3
sandomierski	7,4	7,4	1,9	0,2	0,3	10,5	4
sanocki	87,3	54,2	1,1	0,2	0,2	5,3	1
sejneński	59,1	42,8	4,3	0,0	0,0	8,7	2
sępoleński	77,8	24,8	2,6	0,1	0,2	14,3	2
siedlecki	24,5	18,7	0,3	0,0	0,0	5,6	4
siemiatycki	21,4	34,6	0,5	0,1	0,2	2,8	3
sieradzki	24,6	18,9	0,4	0,0	0,3	19,5	3
sierpecki	50,0	13,8	0,6	0,0	0,1	3,6	3
skarżyski	73,4	58,2	0,4	0,2	0,3	12,2	1
skierniewicki	25,2	22,1	0,3	0,1	0,1	21,9	3
sławieński	15,0	29,1	4,6	0,2	0,1	14,8	2
słubicki	38,9	48,5	1,8	0,2	0,3	3,8	2
słupecki	48,1	15,2	2,5	0,1	0,2	4,8	3
słupski	22,8	36,8	4,5	0,2	0,3	21,5	1
sochaczewski	34,3	14,9	2,2	0,1	0,2	11,3	3
sokołowski	40,9	22,1	0,7	0,0	0,1	13,7	3
sokólski	24,8	25,8	0,2	0,1	0,1	3,1	4
stalowowolski	11,7	52,3	1,5	0,2	0,5	7,5	2
starachowicki	90,7	46,6	1,9	0,1	0,5	8,6	1
stargardzki	10,7	25,2	3,7	0,2	0,4	30,1	2
starogardzki	42,5	43,4	2,6	0,1	0,2	8,3	2
staszowski	38,6	27,8	1,3	0,1	0,2	7,7	3
strzelecki	45,9	41,8	0,3	0,4	0,5	6,7	1
strzelecko-drezdenecki	72,8	51,5	2,9	0,2	0,1	5,5	1
strzeliński	14,6	9,0	0,7	0,2	0,4	3,5	4
strzyżowski	46,5	28,1	0,9	0,1	0,2	5,4	3
sulęciński	51,5	57,3	1,8	0,1	0,2	12,6	1
suski	41,7	48,8	1,3	0,1	0,1	7,9	2
suwalski	59,5	18,0	4,6	0,0	0,0	7,3	2
szamotulski	21,9	31,7	1,6	0,2	0,4	12,6	2
szczecinecki	28,9	46,3	5,1	0,1	0,5	11,8	1
szczycieński	44,3	51,3	3,8	0,1	0,1	6,3	2
sztumski	22,4	17,4	1,8	0,1	0,2	10,5	3
szydłowiecki	39,4	33,7	0,3	0,1	0,5	8,8	2
średzki	0,7	13,1	1,1	0,3	0,3	4,0	4
średzki	0,8	14,8	1,2	0,4	0,3	4,5	4
śremski	15,1	20,2	2,1	0,4	1,5	25,3	1

świdnicki (dolnośląskie region)	7,4	14,0	0,7	0,4	1,2	23,8	2
świdnicki (lubelskie region)	11,8	22,1	1,1	0,6	1,9	37,6	1
świdwiński	15,2	36,1	1,2	0,1	0,2	16,7	3
świebodziński	32,3	43,3	2,3	0,2	0,2	6,5	2
świecki	48,2	36,5	2,9	0,1	0,2	23,8	1
tarnobrzeski	0,0	34,0	1,3	0,2	0,3	8,1	3
tarnogórski	0,9	51,0	1,5	0,8	1,9	23,4	1
tarnowski	74,8	21,9	1,0	0,2	0,1	10,5	2
tatrzański	92,8	47,8	1,7	0,2	0,4	3,0	1
tczewski	12,7	15,0	2,3	0,2	0,9	11,6	3
tomaszowski (lubelskie region)	10,5	22,2	0,3	0,1	0,2	3,7	4
tomaszowski (mazowieckie region)	15,2	32,2	0,4	0,1	0,2	5,4	3
toruński	35,3	34,8	2,1	0,1	0,1	7,2	3
trzebnicki	24,0	26,7	0,6	0,2	0,3	20,4	2
tucholski	64,9	49,8	2,5	0,1	0,1	23,4	1
turecki	34,6	23,9	1,2	0,1	0,5	4,1	3
wadowicki	11,5	24,0	1,7	0,2	0,4	17,3	3
wałbrzyski	35,6	42,3	0,5	0,3	0,5	33,0	1
wałecki	51,7	56,5	3,4	0,2	0,2	6,4	1
warszawski zachodni	46,7	26,0	0,8	0,1	0,8	23,4	2
wąbrzeski	22,7	8,2	1,8	0,1	0,1	8,4	4
wągrowiecki	14,1	19,5	2,2	0,1	0,2	8,4	3
wejherowski	45,8	44,7	2,3	0,1	0,3	23,3	1
węgorzewski	66,6	21,2	13,5	0,1	0,1	10,4	1
węgrowski	38,0	26,9	0,7	0,0	0,1	15,1	3
wielicki	0,3	16,3	1,0	0,2	0,7	14,8	3
wieluński	19,8	24,9	0,6	0,1	0,2	14,4	3
wieruszowski	39,6	26,5	0,3	0,1	0,2	8,1	3
włocławski	16,7	18,7	2,9	0,1	0,1	3,5	4
włodawski	40,7	41,1	1,8	0,1	0,1	3,0	3
włoszczowski	47,9	41,7	0,3	0,0	0,1	3,4	3
wodzisławski	2,2	9,7	1,8	0,7	1,8	8,4	2
wolsztyński	64,3	31,1	3,6	0,4	0,5	10,0	1
wołomiński	20,8	28,3	0,8	0,3	0,5	22,4	2
wołowski	11,8	35,3	1,1	0,2	0,7	3,9	3
wrocławski	10,9	11,0	1,7	0,3	0,5	9,2	3
wrzesiński	23,1	19,0	0,5	0,3	0,8	5,8	3
wschowski	22,5	40,0	2,3	0,2	0,3	8,3	2
wysokomazowiecki	2,5	18,6	0,3	0,0	0,1	5,0	4
wyszkowski	0,1	33,7	1,5	0,1	0,1	4,9	4
zambrowski	0,4	29,9	0,2	0,0	0,1	0,5	4
zamojski	27,7	23,2	0,6	0,1	0,1	4,7	4
zawierciański	33,4	30,5	0,4	0,1	0,5	6,8	3
ząbkowicki	15,8	20,4	1,2	0,5	0,7	11,0	2
zduńskowolski	7,3	21,6	0,6	0,1	0,7	26,0	3
zgierski	14,4	19,0	0,3	0,3	0,6	27,7	2
zgorzelecki	0,1	49,4	0,7	0,3	0,6	5,6	3
zielonogórski	24,9	60,2	2,1	0,3	0,3	15,8	1
złotoryjski	7,1	20,7	0,4	0,2	0,2	12,8	3
złotowski	31,7	47,8	1,5	0,1	0,2	8,3	2
zwoleński	11,0	15,4	0,5	0,1	0,1	15,2	4

żagański	26,4	48,4	0,6	0,2	0,4	8,9	2
żarski	44,7	55,5	0,4	0,3	1,2	8,6	1
żniński	10,5	17,4	4,0	0,2	0,3	17,0	3
żuromiński	73,9	21,1	0,3	0,1	0,1	4,3	3
żyrardowski	60,7	23,1	0,4	0,1	0,7	24,2	2
żywiecki	52,1	52,3	2,5	0,1	0,3	10,4	1

Source: own calculations based on Polish Central Statistical Office data
