# Supplementary material for: Geographic differentiation of agritourism activities in Poland vs. cultural and natural attractiveness of destinations at district level
Source: PLoS One. 2019 Sep 20;14(9):e0222576. doi: 10.1371/journal.pone.0222576 (PMC6754142; doi:10.1371/journal.pone.0222576)
Supplement: S2 Table — Source: own calculations based on Polish Central Statistical Office data. (PDF) [file pone.0222576.s002.pdf]

S2 Table. Diagnostic features used to formulate the Hellwig's synthetic indicator of cultural attractiveness

District	Total number of facilities entered to the register of monuments / 100 km^2	Turists per 10,000 population	Total number of cultural and sport events per 10,000 population	Total number of participants to major events per 1,000 population	Class
aleksandrowski	29,9	299,2	0,9	336,6	2
augustowski	5,9	767,8	1,5	381,3	3
bartoszycki	21,6	250,8	0,3	71,6	3
bełchatowski	5,6	226,9	1,2	943,9	2
będziński	26,9	4 365,4	0,5	186,7	3
bialski	15,6	448,4	0,7	143,1	3
białobrzeski	8,8	0,0	0,0	29,8	4
białogardzki	11,5	428,7	0,8	106,7	3
białostocki	10,4	10 561,5	0,8	172,0	3
bielski	8,7	1 116,8	0,4	107,6	3
bielski	27,9	1 116,8	0,4	107,6	3
bieruńsko-lędziński	18,4	0,0	0,8	129,7	3
bieszczadzki	6,4	118,6	1,8	246,3	3
biłgorajski	8,0	534,8	0,6	95,1	3
bocheński	28,0	8 463,7	0,6	56,6	3
bolesławiecki	17,3	4 541,7	0,8	160,6	3
braniewski	19,2	35 683,4	0,5	71,8	2
brodnicki	8,8	5 465,7	0,5	82,5	3
brzeski (małopolskie region)	21,3	1 592,4	0,6	296,8	3
brzeski (opolskie region)	57,4	1 592,4	0,6	296,8	2
brzeziński	30,6	2 824,9	0,3	32,4	3
brzozowski	27,5	622,5	0,3	60,6	3
buski	13,2	954,5	0,0	0,0	4
bydgoski	9,0	194,9	0,4	90,3	3
bytowski	4,6	6 104,0	3,8	756,5	1
chełmiński	31,7	2 224,1	0,4	57,5	3
chełmski	8,5	0,0	0,3	114,3	3
chodzieski	15,8	0,0	1,7	400,5	2
chojnicki	6,7	600,6	4,5	662,4	1
choszczeński	15,7	0,0	0,0	0,0	3
chrzanowski	22,4	6 955,3	0,6	135,1	3
ciechanowski	24,1	5 179,5	0,3	59,2	3
cieszyński	42,3	2 905,8	1,4	330,5	2
czarnkowsko-trzcianecki	11,2	395,3	1,5	340,5	3
częstochowski	8,0	603,6	0,6	183,8	3
człuchowski	4,1	5 857,8	0,4	91,6	3
dąbrowski	10,0	3 206,0	0,3	101,1	3
dębicki	18,4	1 712,6	0,5	304,3	3
drawski	8,6	0,0	0,0	0,0	4
działdowski	16,9	2 783,6	0,6	300,4	3
dzierżoniowski	56,4	315,2	1,2	607,0	1
elbląski	27,3	0,0	1,2	170,8	2
ełcki	15,5	4 882,7	1,4	430,9	2
garwoliński	18,0	107,4	0,6	66,6	3

gdański	18,4	0,0	0,1	47,0	3
giżycki	26,5	0,0	2,1	499,5	2
gliwicki	27,3	796,6	0,6	140,3	3
głogowski	33,9	2 335,7	2,7	693,5	1
głubczycki	45,3	486,1	0,0	0,0	3
gnieźnieński	33,2	10 996,4	3,0	297,9	1
goleniowski	12,2	0,0	0,2	75,1	3
golubsko-dobrzyński	10,1	8 571,6	0,7	83,9	3
gołdapski	19,4	0,0	0,7	247,4	3
gorlicki	36,9	5 361,3	1,2	223,9	2
gorzowski	10,4	1 579,6	1,7	1 667,2	1
gostyniński	0,0	0,0	0,2	43,9	4
gostyński	37,3	687,3	1,2	163,9	2
górowski	26,7	0,0	0,3	70,3	3
grajewski	11,6	0,0	2,9	239,4	2
grodziski (mazowieckie region)	43,1	404,9	0,5	97,6	2
grodziski (wielkopolskie region)	34,1	404,9	0,5	97,6	3
grójecki	20,6	1 386,7	0,7	125,8	3
grudziądzki	30,5	0,0	1,0	176,5	2
gryficki	28,1	849,5	0,2	16,4	3
gryfiński	25,9	904,7	0,6	231,1	3
hajnowski	8,0	3 844,4	0,9	209,5	3
hrubieszowski	18,4	1 263,4	0,8	177,4	3
iławski	19,6	0,0	1,6	588,2	2
inowrocławski	22,8	1 268,4	2,8	505,0	1
janowski	6,7	899,3	0,6	86,2	3
jarociński	31,9	3 812,4	0,8	931,5	2
jarosławski	49,2	275,4	1,3	273,1	2
jasielski	16,8	4 209,9	0,9	191,6	3
jaworski	63,2	13 122,7	1,0	117,9	1
jeleniogórski	55,7	13 572,5	0,9	210,0	1
jędrzejowski	16,9	1 841,1	1,2	184,6	3
kaliski	12,2	1 490,5	2,0	450,9	2
kamiennogórski	56,8	1 907,7	0,2	29,4	2
kamieński	20,3	10 172,6	0,6	190,6	2
kartuski	11,8	2 685,4	0,6	385,2	3
kazimierski	16,1	0,0	0,6	73,4	3
kędzierzyńsko-kozielski	34,1	313,8	1,0	683,3	2
kępiński	17,6	569,4	1,8	116,9	2
kętrzyński	43,7	5 392,3	1,1	154,2	2
kielecki	15,5	11 203,4	0,5	189,6	3
kluczborski	34,2	1 170,6	5,0	681,4	1
kłobucki	4,7	0,0	0,2	29,4	4
kłodzki	44,6	10 519,4	0,9	117,5	2
kolbuszowski	17,1	6 412,8	0,5	136,1	3
kolneński	8,8	0,0	0,0	0,0	4
kolski	20,0	4 202,4	0,7	116,1	3
kołobrzeski	17,7	15 810,8	6,9	3 165,8	1
konecki	6,5	186,3	0,5	98,0	3
koniński	15,5	80,6	0,5	84,9	3

koszaliński	13,3	0,0	1,4	234,4	3
kościański	60,1	914,0	1,1	194,4	2
kościerski	8,1	25 008,2	1,0	86,2	2
kozienicki	8,1	1 954,8	0,8	479,7	3
krakowski	44,1	5 545,6	0,3	66,1	2
krapkowicki	26,2	0,0	0,6	112,0	3
krasnostawski	20,2	398,2	0,3	16,7	3
kraśnicki	10,8	30,2	0,8	206,7	3
krośnieński	21,4	17 373,7	0,2	36,0	3
krośnieński	27,7	17 373,7	0,2	36,0	3
krotoszyński	22,1	946,4	0,4	105,5	3
kutnowski	24,0	5 275,8	0,5	148,5	3
kwidzyński	26,3	2 980,4	3,5	685,0	1
legionowski	20,5	1 791,7	1,7	293,4	2
legnicki	38,2	2 257,0	0,2	37,1	3
leski	9,7	1 839,6	0,4	48,8	3
leszczyński	39,1	0,0	1,3	276,6	2
leżajski	16,8	1 902,5	0,3	87,7	3
lęborski	7,4	2 211,8	0,5	197,8	3
lidzbarski	33,0	11 517,3	0,5	124,0	2
limanowski	12,5	651,7	0,5	82,8	3
lipnowski	17,0	0,0	0,5	124,9	3
lipski	8,4	0,0	0,0	0,0	4
lubaczowski	18,4	2 313,5	0,9	399,2	3
lubański	48,4	533,1	0,7	159,7	2
lubartowski	8,8	33 259,0	0,4	140,2	2
lubelski	15,7	340,7	0,5	181,4	3
lubiński	31,5	0,0	6,7	4 987,7	1
lubliniecki	11,1	0,0	0,4	104,2	3
lwówecki	47,0	2 886,2	1,5	474,7	1
łańcucki	59,1	56 694,6	0,7	101,8	1
łaski	5,0	296,5	0,4	89,6	3
łęczycki	16,6	6 621,5	0,0	0,0	3
łęczyński	14,1	0,0	3,5	970,6	1
łobeski	21,6	55,2	0,8	97,2	3
łomżyński	7,3	5 826,2	0,6	117,1	3
łosicki	19,7	1,3	1,0	206,6	3
łowicki	22,3	29 032,9	1,5	276,8	1
łódzki wschodni	3,4	0,0	1,4	153,6	3
łukowski	8,1	2 070,5	0,5	74,0	3
makowski	5,2	98,5	0,7	90,7	3
malborski	31,4	99 964,5	1,7	718,9	1
miechowski	12,3	1 214,6	0,4	91,1	3
mielecki	15,2	766,7	2,1	740,0	2
międzychodzki	31,7	2 295,3	2,4	845,2	1
międzyrzecki	26,9	10 286,8	0,3	85,7	3
mikołowski	22,3	791,2	0,5	229,1	3
milicki	11,0	0,0	0,8	94,3	3
miński	14,4	1 442,0	0,3	42,3	3
mławski	14,0	921,5	0,7	196,1	3
mogileński	10,4	648,3	0,4	93,2	3
moniecki	4,6	0,0	0,5	85,2	3

mrągowski	26,8	2 999,7	2,0	1 029,0	1
myszkowski	8,4	0,0	0,6	370,5	3
myślenicki	23,5	2 704,9	0,3	109,5	3
myśliborski	17,5	415,2	0,4	106,3	3
nakielski	12,5	1 242,4	0,5	61,1	3
namysłowski	26,6	0,0	0,7	124,2	3
nidzicki	12,5	0,0	0,9	239,9	3
niżański	13,6	0,0	1,9	309,8	2
nowodworski (mazowieckie region)	19,7	2 775,4	0,5	86,1	3
nowodworski (pomorskie region)	19,6	2 775,4	0,5	86,1	3
nowomiejski	17,9	0,0	1,4	340,3	2
nowosądecki	26,2	1 358,3	3,1	437,4	1
nowosolski	85,2	644,1	0,3	103,5	2
nowotarski	28,6	15 526,3	0,4	81,2	2
nowotomyski	19,6	1 149,8	0,7	121,6	3
nyski	48,2	850,3	2,0	246,5	1
obornicki	22,1	2 374,3	0,7	130,7	3
olecki	19,6	0,0	2,0	260,3	2
oleski	9,0	1 469,4	0,0	0,0	4
oleśnicki	19,4	1 079,2	1,6	220,9	2
olkuski	14,9	832,1	0,5	64,9	3
olsztyński	35,6	4 731,0	0,4	79,5	2
oławski	24,6	0,0	2,2	281,9	2
opatowski	17,9	188,2	0,2	37,6	3
opoczyński	9,3	753,1	0,3	22,1	3
opolski (lubelskie region)	15,6	189,7	0,8	183,8	3
opolski (opolskie region)	14,1	189,7	0,8	183,8	3
ostrołęcki	3,9	1 570,9	0,3	65,3	4
ostrowiecki	22,5	6 463,6	6,4	1 354,8	1
ostrowski (mazowieckie region)	7,1	2,9	1,2	242,4	3
ostrowski (wielkopolskie region)	12,9	2,9	1,2	242,4	3
ostródzki	25,9	14 141,4	3,4	634,3	1
ostrzeszowski	10,2	1 172,1	0,7	90,5	3
oświęcimski	37,7	139 063,2	2,7	857,1	1
otwocki	21,6	1 945,2	0,6	113,3	3
pabianicki	10,0	980,9	0,4	82,2	3
pajęczański	5,6	0,0	0,8	119,5	3
parczewski	11,0	289,0	0,3	85,0	3
piaseczyński	52,5	446,6	0,1	24,9	2
pilski	16,7	2 383,1	2,9	826,3	1
pińczowski	14,7	1 675,5	0,5	50,5	3
piotrkowski	8,7	350,7	0,5	69,1	3
piski	9,5	4 564,2	0,4	70,0	3
pleszewski	17,0	17 163,6	0,8	253,9	2
płocki	11,1	1 129,1	0,3	48,6	3
płoński	15,3	0,0	2,4	364,5	2
poddębicki	6,0	587,0	0,5	65,2	3
policki	16,4	198,6	0,4	69,5	3

polkowicki	26,7	0,0	9,5	1 018,8	1
poznański	30,8	4 859,3	1,0	197,8	2
proszowicki	24,1	326,2	0,5	75,7	3
prudnicki	57,0	3 494,1	0,9	118,3	2
pruszkowski	71,1	2 795,1	0,9	212,3	1
przasnyski	8,3	1 581,6	0,9	139,8	3
przemyski	34,7	0,0	0,1	20,2	3
przeworski	40,8	1 243,6	0,4	110,7	2
przysuski	7,1	661,3	1,4	205,5	3
pszczyński	29,9	26 651,2	0,5	139,4	2
pucki	29,0	22 677,4	1,4	3 446,5	1
puławski	48,2	13 244,0	3,9	765,9	1
pułtuski	14,6	1 497,6	1,0	90,8	3
pyrzycki	22,6	0,0	0,8	115,2	3
raciborski	25,2	1 708,3	0,8	184,1	3
radomski	9,9	1 036,5	0,8	114,7	3
radomszczański	8,2	1 335,9	0,3	65,4	3
radziejowski	13,7	0,0	1,9	156,2	2
radzyński	15,6	0,0	0,3	58,5	3
rawicki	68,2	321,6	2,2	182,2	1
rawski	21,2	510,0	0,8	265,2	3
ropczycko-sędziszowski	19,5	0,0	0,4	162,1	3
rybnicki	63,8	0,0	0,4	142,9	2
rycki	27,3	7 284,8	0,5	140,8	3
rypiński	11,3	939,2	0,0	0,0	4
rzeszowski	41,1	30,0	0,4	69,3	3
sandomierski	30,3	10 237,8	3,9	348,8	1
sanocki	23,8	23 503,0	1,1	304,7	2
sejneński	17,1	3 824,9	2,0	113,3	2
sępoleński	12,0	0,0	0,5	96,7	3
siedlecki	11,1	276,9	0,5	85,8	3
siemiatycki	9,6	2 081,4	3,5	399,9	2
sieradzki	11,9	1 537,2	0,6	102,7	3
sierpecki	9,2	14 832,3	0,2	57,1	3
skarżyski	5,6	8 293,3	0,1	19,7	3
skierniewicki	14,1	856,8	0,0	0,0	3
sławieński	12,6	17 467,3	0,4	56,3	3
słubicki	7,2	53,1	1,1	257,1	3
słupecki	17,7	607,1	1,0	168,0	3
słupski	10,8	8 036,2	0,3	299,3	3
sochaczewski	19,2	28 040,0	0,5	132,7	2
sokołowski	11,5	11 591,8	0,4	36,6	3
sokólski	7,3	626,3	1,0	213,6	3
stalowowolski	11,3	10 558,1	1,8	329,6	2
starachowicki	14,9	2 039,8	0,8	214,0	3
stargardzki	20,6	2 340,9	1,7	193,1	2
starogardzki	20,5	697,7	0,9	215,6	3
staszowski	16,6	0,0	1,1	181,0	3
strzelecki	26,9	652,8	0,4	106,6	3
strzelecko-drezdenecki	12,3	1 465,4	0,8	263,2	3
strzeliński	34,6	0,0	0,7	50,0	3
strzyżowski	27,8	1 030,0	0,0	0,0	3

sulęciński	7,2	633,9	0,8	311,0	3
suski	10,5	2 199,1	0,1	29,7	3
suwalski	8,2	0,0	2,0	219,0	3
szamotulski	19,3	2 069,0	0,6	59,6	3
szczecinecki	8,3	1 572,0	0,6	125,2	3
szczycieński	14,3	1 147,0	0,9	159,2	3
sztumski	19,8	1 360,1	0,0	0,0	3
szydłowiecki	15,0	6 055,6	0,0	0,0	3
średzki	42,8	676,6	0,6	374,4	2
średzki	24,7	676,6	0,6	374,4	2
śremski	35,9	375,2	0,5	106,1	3
świdnicki (dolnośląskie region)	60,6	6 848,6	0,9	298,8	1
świdnicki (lubelskie region)	20,9	6 848,6	0,9	298,8	2
świdwiński	13,2	0,0	0,0	0,0	4
świebodziński	37,0	1 898,9	2,0	142,4	2
świecki	12,1	0,0	1,4	187,0	3
tarnobrzeski	9,2	8 403,9	0,4	48,7	3
tarnogórski	30,4	597,8	0,3	72,6	3
tarnowski	28,3	1 580,9	1,8	484,2	2
tatrzański	66,7	32 949,3	1,9	1 446,8	1
tczewski	32,4	5 553,2	0,8	158,9	2
tomaszowski (lubelskie region)	12,8	5 131,5	0,4	61,4	3
tomaszowski (mazowieckie region)	11,3	5 131,5	0,4	61,4	3
toruński	9,8	1 045,5	0,4	47,6	3
trzebnicki	18,6	95,0	0,8	356,1	3
tucholski	7,3	1 499,8	0,2	51,6	4
turecki	15,6	947,3	0,6	86,7	3
wadowicki	39,0	14 822,4	0,8	231,5	2
wałbrzyski	39,1	0,0	0,2	26,5	3
wałecki	16,0	2 863,3	1,7	259,6	2
warszawski zachodni	18,9	14,8	1,1	270,2	3
wąbrzeski	16,5	0,0	0,0	0,0	3
wągrowiecki	21,3	569,5	3,1	331,5	2
wejherowski	14,1	2 675,8	0,8	139,1	3
węgorzewski	22,5	4 696,2	0,9	241,5	2
węgrowski	11,7	4 819,2	0,6	64,6	3
wielicki	42,6	140 014,3	2,0	397,8	1
wieluński	8,4	2 536,4	0,5	122,0	3
wieruszowski	4,5	0,0	0,5	94,8	3
włocławski	17,8	2 528,3	0,3	28,9	3
włodawski	6,0	19 247,8	0,8	82,0	3
włoszczowski	9,7	0,0	1,1	208,4	3
wodzisławski	31,7	363,8	0,3	89,3	3
wolsztyński	26,8	4 286,8	0,2	17,4	3
wołomiński	10,4	454,6	0,5	84,1	3
wołowski	25,3	0,0	1,5	440,3	2
wrocławski	29,7	731,8	1,6	153,2	2
wrzesiński	22,9	1 857,3	0,9	347,1	2
wschowski	44,6	2 152,4	0,8	209,0	2

wysokomazowiecki	14,6	5 654,8	0,7	242,5	3
wyszkowski	10,0	0,0	0,8	248,6	3
zambrowski	10,2	0,0	0,9	167,8	3
zamojski	14,4	0,0	0,4	37,2	3
zawierciański	11,8	0,0	0,8	209,1	3
ząbkowicki	53,2	352,7	0,8	90,6	2
zduńskowolski	8,1	1 185,2	0,6	134,1	3
zgierski	14,5	677,8	0,6	135,5	3
zgorzelecki	41,0	473,0	1,0	189,8	2
zielonogórski	23,8	3 492,2	0,4	50,4	3
złotoryjski	42,9	0,0	0,5	67,8	2
złotowski	8,7	474,9	0,6	75,9	3
zwoleński	8,9	6 601,7	0,0	0,0	3
żagański	35,6	1 531,7	0,4	37,3	3
żarski	40,3	567,4	0,6	97,4	2
żniński	13,3	53 727,1	0,3	41,1	2
żuromiński	7,4	524,9	0,3	380,5	3
żyrardowski	53,5	690,3	0,7	107,4	2
żywiecki	16,1	8 317,3	1,0	357,1	2

Source: own calculations based on Polish Central Statistical Office data
