# Supplementary material for: Geographic differentiation of agritourism activities in Poland vs. cultural and natural attractiveness of destinations at district level
Source: PLoS One. 2019 Sep 20;14(9):e0222576. doi: 10.1371/journal.pone.0222576 (PMC6754142; doi:10.1371/journal.pone.0222576)
Supplement: S3 Table — Source: own calculation based on data of Polish Central Statistical Office and Agency for Restructuring and Modernization of Agriculture. (PDF) [file pone.0222576.s003.pdf]

S3 Table. Variables for Pearson's linear correlation coefficient for the phenomena under consideration

District	V1 Cultural attractiveness (size of the synthetic indicator)	V2 Natural attractiveness (size of the synthetic indicator)	V3 Eligible costs of investments for agritourism supported by the EU funds (PLN/ km^2)	V3 Number of beds in agricultural accommodation facilities per 100 km^2
aleksandrowski	0,1147	0,0661	0	0,00
augustowski	0,0961	0,1599	4420	3,19
bartoszycki	0,0751	0,0833	19859	0,83
bełchatowski	0,1133	0,1046	334	5,17
będziński	0,0993	0,2369	0	0,00
bialski	0,0797	0,0407	3238	2,43
białobrzeski	0,0446	0,0928	446	0,00
białogardzki	0,0743	0,0764	237	0,00
białostocki	0,0917	0,1071	915	2,86
brzeski (małopolskie region)	0,0596	0,0116	2439	0,00
brzeski (opolskie region)	0,0879	0,1313	19438	0,00
bieruńsko-lędziński	0,0855	0,1839	0	0,00
bieszczadzki	0,0957	0,1823	2000	10,89
biłgorajski	0,0625	0,0439	1223	3,57
bocheński	0,1041	0,1735	2402	1,54
bolesławiecki	0,0919	0,1193	1000	1,15
braniewski	0,1332	0,1533	1992	1,05
brodnicki	0,0698	0,1378	4938	2,50
brzeski	0,0962	0,1484	6008	0,00
brzeski	0,1435	0,0907	4053	0,00
brzeziński	0,0901	0,1352	0	0,00
brzozowski	0,0826	0,1290	1792	2,23
buski	0,0516	0,1060	3334	2,07
bydgoski	0,0594	0,1651	994	2,72
bytowski	0,1685	0,1455	1375	7,76
chełmiński	0,0932	0,1483	996	4,36
chełmski	0,0549	0,0646	1549	0,00
chodzieski	0,1151	0,1256	1834	1,66
chojnicki	0,1691	0,2208	3917	6,16
choszczeński	0,0535	0,1682	1037	1,13
chrzanowski	0,0992	0,2424	0	0,00
ciechanowski	0,0867	0,0918	1533	0,00
cieszyński	0,1490	0,2231	3407	29,59
czarnkowsko-trzcianecki	0,1009	0,1516	817	10,60
częstochowski	0,0670	0,0648	1039	5,13
człuchowski	0,0595	0,1155	2027	0,00
dąbrowski	0,0644	0,0224	1613	0,00
dębicki	0,0891	0,0649	614	1,29
drawski	0,0429	0,2234	745	3,77
działdowski	0,0909	0,1158	608	1,91
dzierżoniowski	0,1695	0,1652	2270	5,22
elbląski	0,1097	0,2196	1269	4,76
ełcki	0,1193	0,1501	2775	5,54

garwoliński	0,0746	0,0998	612	4,67
gdański	0,0622	0,1368	363	14,36
giżycki	0,1461	0,2538	2960	10,76
gliwicki	0,0945	0,1264	1174	1,66
głogowski	0,1840	0,1229	2164	0,00
głubczycki	0,0943	0,0339	1424	1,63
gnieźnieński	0,1855	0,0817	1763	2,10
goleniowski	0,0583	0,1356	360	2,85
golubsko-dobrzyński	0,0812	0,0971	1860	8,97
gołdapski	0,0903	0,1299	1643	1,73
gorlicki	0,1354	0,1501	2138	3,42
gorzowski	0,1643	0,1809	2470	3,95
gostyniński	0,0371	0,1004	5275	0,00
gostyński	0,1238	0,0894	4005	3,12
górowski	0,0803	0,1476	1261	0,00
grajewski	0,1266	0,0490	2038	2,27
grodziski (mazowieckie region)	0,1110	0,1511	1138	0,00
grodziski (wielkopolskie region)	0,0994	0,0801	651	0,00
grójecki	0,0878	0,0432	1251	1,18
grudziądzki	0,1091	0,1206	1177	0,00
gryficki	0,0780	0,0726	560	1,82
gryfiński	0,0972	0,1527	96	3,09
hajnowski	0,0820	0,2411	1527	15,27
hrubieszowski	0,0884	0,0255	724	1,18
iławski	0,1285	0,1650	1068	5,52
inowrocławski	0,1578	0,1020	841	5,71
janowski	0,0622	0,1497	1049	4,00
jarociński	0,1508	0,1026	2924	2,24
jarosławski	0,1475	0,1039	1087	3,11
jasielski	0,0948	0,1212	240	3,61
jaworski	0,1711	0,1440	345	2,75
jeleniogórski	0,1671	0,2003	3156	18,18
jędrzejowski	0,0971	0,1009	875	1,11
kaliski	0,1229	0,0536	7895	8,39
kamiennogórski	0,1180	0,1112	35585	25,76
kamieński	0,1045	0,2002	865	2,90
kartuski	0,0868	0,1670	4980	18,82
kazimierski	0,0729	0,0052	1749	0,00
kędzierzyńsko-kozielski	0,1411	0,1291	674	0,00
kępiński	0,1067	0,0577	2245	0,00
kętrzyński	0,1385	0,0880	1496	1,62
kielecki	0,0961	0,1448	3193	13,22
kluczborski	0,2243	0,1324	821	3,76
kłobucki	0,0443	0,0391	1750	3,49
kłodzki	0,1413	0,1669	2322	20,10
kolbuszowski	0,0862	0,1097	760	0,00
kolneński	0,0433	0,0437	6019	1,06
kolski	0,0909	0,0501	2427	4,31
kołobrzeski	0,3535	0,1616	2331	2,48
konecki	0,0572	0,1436	163	0,00

koniński	0,0716	0,1176	3655	13,30
koszaliński	0,0954	0,1266	1502	4,48
kościański	0,1527	0,0857	1687	0,00
kościerski	0,1129	0,2037	2280	9,78
kozienicki	0,0895	0,1019	334	1,64
krakowski	0,1145	0,1395	1667	6,26
krapkowicki	0,0906	0,1675	158	2,71
krasnostawski	0,0697	0,0520	744	0,00
kraśnicki	0,0774	0,0680	2733	0,00
krośnieński	0,0983	0,1470	949	2,16
krośnieński	0,1075	0,2029	1329	3,02
krotoszyński	0,0800	0,1079	3226	1,49
kutnowski	0,0959	0,0213	772	0,00
kwidzyński	0,1899	0,1459	3799	3,95
legionowski	0,1217	0,3624	4627	3,85
legnicki	0,0955	0,0949	270	4,03
leski	0,0599	0,2015	3603	37,49
leszczyński	0,1326	0,1630	1362	5,03
leżajski	0,0704	0,1164	362	0,00
lęborski	0,0661	0,1837	3009	4,39
lidzbarski	0,1175	0,0806	1579	7,51
limanowski	0,0659	0,1384	2249	6,83
lipnowski	0,0734	0,0728	880	1,57
lipski	0,0426	0,0341	970	0,00
lubaczowski	0,1043	0,1449	1622	1,38
lubański	0,1258	0,1588	1748	10,98
lubartowski	0,1156	0,0633	2521	1,78
lubelski	0,0751	0,0264	5011	4,17
lubiński	0,3644	0,1257	246	0,00
lubliniecki	0,0618	0,1415	443	0,00
lwówecki	0,1651	0,0926	3263	3,38
łańcucki	0,2275	0,0712	1933	2,43
łaski	0,0524	0,0976	497	0,00
łęczycki	0,0664	-0,0025	323	0,00
łęczyński	0,1776	0,0782	1990	4,87
łobeski	0,0877	0,1103	751	0,00
łomżyński	0,0718	0,0492	6935	2,21
łosicki	0,0941	0,0629	825	4,02
łowicki	0,1659	0,0256	167	4,05
łódzki wschodni	0,0766	0,0994	1504	6,80
łukowski	0,0612	0,0394	568	0,00
makowski	0,0587	0,0134	1406	1,41
malborski	0,2946	0,0832	2288	2,43
miechowski	0,0656	0,0925	2618	0,00
mielecki	0,1403	0,0881	315	0,00
międzychodzki	0,1827	0,2700	9716	6,61
międzyrzecki	0,1014	0,1694	1235	7,78
mikołowski	0,0895	0,2188	0	0,00
milicki	0,0717	0,1719	1100	0,00
miński	0,0632	0,0950	291	3,18
mławski	0,0798	0,0817	256	0,00
mogileński	0,0625	0,0794	2414	0,00

moniecki	0,0531	0,0493	1084	0,00
mrągowski	0,1748	0,2177	3295	18,52
myszkowski	0,0747	0,0891	1310	7,93
myślenicki	0,0836	0,1147	1745	9,81
myśliborski	0,0739	0,1823	531	0,00
nakielski	0,0659	0,0913	663	4,91
namysłowski	0,0937	0,1661	811	3,07
nidzicki	0,0834	0,1364	204	4,90
niżański	0,1130	0,0627	891	0,00
nowodworski (mazowieckie region)	0,0819	0,1477	3969	1,73
nowodworski (pomorskie region)	0,0818	0,1537	4105	1,79
nowomiejski	0,1077	0,1023	2030	0,00
nowosądecki	0,1660	0,2086	4091	9,88
nowosolski	0,1499	0,1476	392	2,59
nowotarski	0,1145	0,1604	1378	29,78
nowotomyski	0,0847	0,1636	1890	6,33
nyski	0,1631	0,1279	743	1,14
obornicki	0,0911	0,1377	1128	3,45
olecki	0,1217	0,1285	2608	0,53
oleski	0,0462	0,0544	884	6,89
oleśnicki	0,1117	0,0771	65598	0,00
olkuski	0,0708	0,1389	93	9,71
olsztyński	0,1047	0,1549	1458	9,06
oławski	0,1349	0,0689	1427	0,00
opatowski	0,0639	0,0330	595	1,10
opoczyński	0,0531	0,0648	1213	0,00
opolski (lubelskie region)	0,0839	0,0957	8085	0,00
opolski (opolskie region)	0,0817	0,0259	210	0,00
ostrołęcki	0,0501	0,0122	1314	3,81
ostrowiecki	0,2683	0,1685	718	2,59
ostrowski (mazowieckie region)	0,0827	0,0143	4767	0,82
ostrowski (wielkopolskie region)	0,0921	0,0176	5006	1,29
ostródzki	0,2074	0,1831	1778	7,55
ostrzeszowski	0,0702	0,1421	8269	3,55
oświęcimski	0,3546	0,1290	493	0,00
otwocki	0,0881	0,2036	0	0,00
pabianicki	0,0615	0,1288	0	7,11
pajęczański	0,0634	0,0427	1074	1,87
parczewski	0,0586	0,0538	2251	0,00
piaseczyński	0,1070	0,1915	1240	7,25
pilski	0,1687	0,1540	1060	5,89
pińczowski	0,0706	0,1146	1648	2,45
piotrkowski	0,0611	0,0658	813	6,02
piski	0,0648	0,2270	1318	3,26
pleszewski	0,1185	0,1313	2864	3,89
płocki	0,0581	0,1037	392	1,67
płoński	0,1282	0,0655	1235	2,03
poddębicki	0,0554	0,0684	1046	0,00

policki	0,0684	0,1874	121	3,31
polkowicki	0,2623	0,1250	257	0,00
poznański	0,1195	0,1826	3432	14,98
proszowicki	0,0821	0,0081	2897	0,00
prudnicki	0,1439	0,0439	564	0,00
pruszkowski	0,1634	0,2689	1599	15,45
przasnyski	0,0758	0,0259	2647	2,21
przemyski	0,0847	0,1922	3147	4,54
przeworski	0,1062	0,1605	4550	0,00
przysuski	0,0866	0,0863	107	4,49
pszczyński	0,1411	0,1929	1180	5,10
pucki	0,2846	0,1774	1764	11,54
puławski	0,2569	0,1304	8219	5,89
pułtuski	0,0836	0,0452	471	0,00
pyrzycki	0,0887	0,0526	3439	0,00
raciborski	0,1010	0,1904	375	0,00
radomski	0,0723	0,0804	504	0,00
radomszczański	0,0546	0,0960	535	1,39
radziejowski	0,1054	0,0132	1923	2,64
radzyński	0,0651	0,0101	639	0,00
rawicki	0,1846	0,0522	7224	2,98
rawski	0,0968	0,0839	951	0,00
ropczycko-sędziszowski	0,0778	0,0893	1042	2,19
rybnicki	0,1322	0,1826	850	0,00
rycki	0,1043	0,0703	3326	0,00
rypiński	0,0486	0,0702	2377	0,00
rzeszowski	0,1015	0,1016	4329	4,08
sandomierski	0,2018	0,0437	3042	1,78
sanocki	0,1490	0,1825	1370	0,95
sejneński	0,1162	0,1643	2825	10,99
sępoleński	0,0653	0,1695	688	0,00
siedlecki	0,0640	0,0357	839	0,94
siemiatycki	0,1471	0,0674	792	1,17
sieradzki	0,0709	0,0834	548	1,07
sierpecki	0,0767	0,0612	235	2,93
skarżyski	0,0568	0,2064	482	0,00
skierniewicki	0,0527	0,0896	862	1,33
sławieński	0,0906	0,1309	139	4,67
słubicki	0,0799	0,1469	889	6,81
słupecki	0,0915	0,1022	5033	7,95
słupski	0,0828	0,1831	424	3,82
sochaczewski	0,1247	0,0994	596	2,72
sokołowski	0,0786	0,0934	1707	3,18
sokólski	0,0780	0,0512	1310	3,21
stalowowolski	0,1257	0,1271	226	0,00
starachowicki	0,0863	0,1975	2574	6,12
stargardzki	0,1161	0,1540	79	3,00
starogardzki	0,0967	0,1465	1063	6,47
staszowski	0,0918	0,1093	1671	0,00
strzelecki	0,0867	0,1822	895	0,00
strzelecko-drezdenecki	0,0846	0,1888	1694	2,24
strzeliński	0,1004	0,0396	1070	5,31

strzyżowski	0,0727	0,1067	3411	3,17
sulęciński	0,0784	0,1808	230	3,23
suski	0,0542	0,1296	756	12,24
suwalski	0,0999	0,1229	3917	6,81
szamotulski	0,0798	0,1352	9096	8,01
szczecinecki	0,0675	0,1896	391	0,00
szczycieński	0,0831	0,1547	970	5,44
sztumski	0,0621	0,0783	13925	9,17
szydłowiecki	0,0632	0,1293	436	0,00
średzki	0,1257	0,0412	7575	0,00
średzki	0,1010	0,0535	8546	0,00
śremski	0,1008	0,2058	1349	0,00
świdnicki (dolnośląskie region)	0,1636	0,1463	314	4,18
świdnicki (lubelskie region)	0,1113	0,2542	2299	6,62
świdwiński	0,0498	0,1059	2085	3,11
świebodziński	0,1432	0,1450	433	0,00
świecki	0,0922	0,1867	1101	8,21
tarnobrzeski	0,0704	0,0653	831	6,33
tarnogórski	0,0868	0,2702	0	0,00
tarnowski	0,1438	0,1389	370	0,00
tatrzański	0,3071	0,1844	1887	97,25
tczewski	0,1158	0,1041	5099	0,00
tomaszowski (lubelskie region)	0,0704	0,0276	1117	1,68
tomaszowski (mazowieckie region)	0,0682	0,0643	1622	2,44
toruński	0,0586	0,1154	845	1,06
trzebnicki	0,0970	0,1245	600	0,00
tucholski	0,0514	0,2044	777	5,86
turecki	0,0747	0,0974	1591	0,00
wadowicki	0,1462	0,1079	2583	3,87
wałbrzyski	0,0918	0,2176	1739	59,53
wałecki	0,1135	0,1873	1400	10,23
warszawski zachodni	0,1005	0,1728	3723	19,10
wąbrzeski	0,0548	0,0534	2584	0,00
wągrowiecki	0,1524	0,0655	1467	0,00
wejherowski	0,0844	0,1950	1486	10,19
węgorzewski	0,1064	0,2177	2683	13,28
węgrowski	0,0747	0,0985	1523	0,00
wielicki	0,3164	0,0803	487	3,89
wieluński	0,0663	0,0849	2878	3,89
wieruszowski	0,0532	0,0913	723	6,24
włocławski	0,0716	0,0510	842	0,00
włodawski	0,0948	0,1090	3298	3,03
włoszczowski	0,0822	0,1008	860	1,76
wodzisławski	0,0897	0,1550	0	0,00
wolsztyński	0,0826	0,2222	3167	5,26
wołomiński	0,0623	0,1489	469	0,00
wołowski	0,1271	0,1088	2012	0,00
wrocławski	0,1224	0,0862	1110	2,33
wrzesiński	0,1081	0,1098	2944	2,53

wschowski	0,1278	0,1331	212757	0,00
wysokomazowiecki	0,0918	0,0053	2690	0,78
wyszkowski	0,0778	0,0518	107	2,28
zambrowski	0,0765	0,0080	1296	0,00
zamojski	0,0631	0,0589	1814	4,81
zawierciański	0,0792	0,1030	3313	4,99
ząbkowicki	0,1284	0,1450	316	1,50
zduńskowolski	0,0660	0,1045	2491	0,00
zgierski	0,0753	0,1302	234	0,00
zgorzelecki	0,1248	0,1012	1102	0,00
zielonogórski	0,0845	0,1886	301	2,00
złotoryjski	0,1061	0,0664	983	8,85
złotowski	0,0621	0,1319	1106	8,52
zwoleński	0,0548	0,0414	644	2,79
żagański	0,0959	0,1395	325	2,12
żarski	0,1097	0,2240	147	5,74
żniński	0,1405	0,1094	1385	6,90
żuromiński	0,0669	0,0954	1854	1,61
żyrardowski	0,1276	0,1793	177	0,00
żywiecki	0,1134	0,1815	5307	31,15

Source: own calculation based on data of Polish Central Statistical Office and
Agency for Restructuring and Modernization of Agriculture
